# Emerging bioelectrochemical technologies for biogas production and upgrading in cascading circular bioenergy systems

**DOI:** 10.1016/j.isci.2021.102998

**Published:** 2021-08-18

**Authors:** Xue Ning, Richen Lin, Richard O'Shea, David Wall, Chen Deng, Benteng Wu, Jerry D. Murphy

**Affiliations:** 1 MaREI Centre, Environmental Research Institute, School of Engineering, University College Cork, Cork T23XE10, Ireland; 2 Civil, Structural, and Environmental Engineering, School of Engineering and Architecture, University College Cork, Cork T23XE10, Ireland

**Keywords:** Electrochemical energy production, Microbiology, Energy systems

## Abstract

Biomethane is suggested as an advanced biofuel for the hard-to-abate sectors such as heavy transport. However, future systems that optimize the resource and production of biomethane have yet to be definitively defined. This paper assesses the opportunity of integrating anaerobic digestion (AD) with three emerging bioelectrochemical technologies in a circular cascading bioeconomy, including for power-to-gas AD (P2G-AD), microbial electrolysis cell AD (MEC-AD), and AD microbial electrosynthesis (AD-MES). The mass and energy flow of the three bioelectrochemical systems are compared with the conventional AD amine scrubber system depending on the availability of renewable electricity. An energy balance assessment indicates that P2G-AD, MEC-AD, and AD-MES circular cascading bioelectrochemical systems gain positive energy outputs by using electricity that would have been curtailed or constrained (equivalent to a primary energy factor of zero). This analysis of technological innovation, aids in the design of future cascading circular biosystems to produce sustainable advanced biofuels.

## Graphical abstract

**Figure:**
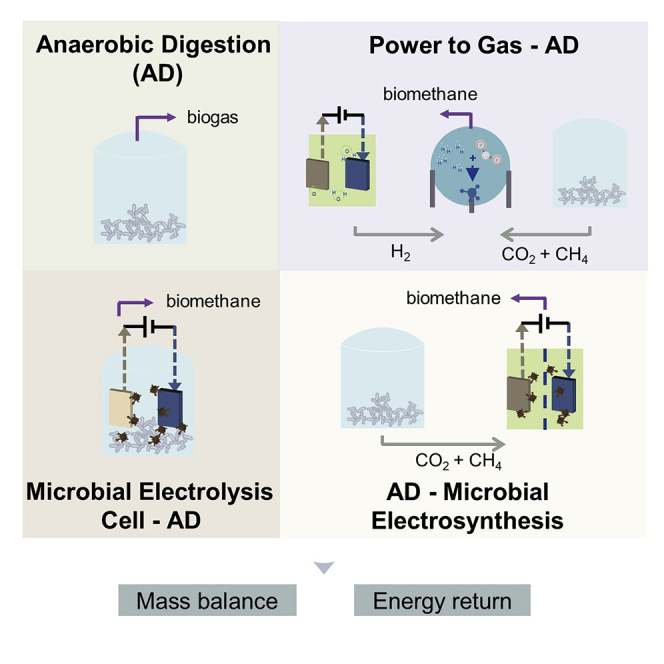

## Introduction

The EU-27 has targeted a complete transition to a sustainable energy landscape by 2050 (Colmenar-Santos et al., 2019) in which all grid electricity will be supplied by renewable energy. However, the hard-to-abate sectors such as freight haulage, airlines, and shipping are not readily electrified. The development of advanced biofuels (such as biomethane) may provide flexibility in the progression of the European economy toward more sustainable use of renewable resources. The EU Renewable Energy Directive mandates a minimum share of advanced biofuels for transport of at least 3.5% in 2030 (Giuntoli, 2018). By 2040, Europe aims to have 10% biomethane in gas grids on a volume basis (IEA, 2020). The International Energy Agency (IEA) has assessed that the full utilization of sustainable biomethane could cover approximately 20% of today's worldwide gas demand (IEA, 2020). Therefore, to maximize the potential of advanced biofuels, a roadmap for technology advances in the production of biofuels must be developed.

Anaerobic digestion (AD) is a proven technology for producing biogas, which can be upgraded to biomethane (aka green gas) as a renewable energy vector. By the end of 2019, there were a total of 18,943 biogas plants and 725 biomethane plants across Europe according to the European Biogas Association Statistical Report 2020 (EBA, 2020). It is predicted that the potential for global biogas will be 50% larger than today by 2040 due to the growing supply of available feedstocks (IEA, 2020); this could enable the production of biomethane for use as an advanced renewable transportation fuel (Ahlström et al., 2020). In addition to biogas production, the digestate produced in AD can be used to return essential nutrients to farmland in the form of organic fertilizer. The authors postulate that a beneficial use of on-farm feedstocks, cultivated marine feedstocks, and municipal wastes is in a sustainable closed-loop system which can generate revenues from the sale of renewable energy and biofertilizer (Allen et al., 2016; Tabassum et al., 2016; Wall et al., 2013). However, most current digesters are implemented as stand-alone AD systems without optimization of circularity in system design. The implementation of conventional AD faces several challenges: the biodegradability of on-farm feedstocks (such as animal slurry, crop straws, and late-cut grass) can be low (less than 50%) in digesters; the energy contained within lignin and cellulose portions of the digestate cannot be fully utilized; and effective and economical approaches to biogas upgrading are challenging (Allen et al., 2015; Pecchi and Baratieri, 2019; Ullah Khan et al., 2017).

The composition of typical raw biogas from AD plants is 60–70% CH_4_, 30–40% CO_2,_ with small amounts of H_2_S, N_2,_ NH_3_, and water vapor (Jönsson et al., 2003). In order to produce biomethane with a high CH_4_ purity, techniques for biogas upgrading are mainly divided into two categories: CO_2_ removal based processes, such as water/amine scrubbing, cryogenic separation, pressure swing adsorption, and membranes (Nguyen et al., 2020); and CO_2_-hydrogenation-based processes, through various methods such as photocatalysis, electrocatalysis, biocatalysis, and heterogeneous catalysis. The combination of biocatalysis and electrocatalysis in bioelectrochemical systems (BESs) powered by renewable electricity sourced from wind turbines or solar PV (Fu et al., 2020) may be used to directly reduce CO_2_ to methane or provide H_2_ which hydrogenates CO_2_ into methane. Compared with CO_2_-removal-based technologies, CO_2_ hydrogenation is advantageous due to the conversion of CO_2_ to CH_4_, resulting in increased biomethane production. The integration of renewable electricity with AD may significantly improve the biomethane yield, whilst achieving simultaneous biogas production and upgrading; however, the economic feasibility of such methods would need to be addressed (Rajendran et al., 2019; Vo et al., 2018). Furthermore, to realize deep sustainability in a circular system with synergistic utilization of by-products and residual streams, the supply of “surplus” renewable electricity would be a significant challenge since curtailed or constrained electricity is typically intermittent.

In a typical BES system, electromethanogenesis is a biocathodic reaction whereby the electrical current and CO_2_ can be converted to methane in the presence of biocatalysts, namely methanogens (Blasco-Gómez et al., 2017; Cheng et al., 2009). BESs can realize this electrical-to-chemical energy conversion as a promising “power to methane” technology for renewable energy storage. Currently, Norway has a 98% share of electrical energy supply acquired from renewable sources, while in Iceland, up to 85% of the total primary energy supply comes from renewable sources. By availing of renewable electricity, synergies between bioelectrochemical and AD technologies can be expected with promising advantages in accelerating the degradation of COD or VFAs, enhancing methanogenesis and biogas production (De Vrieze et al., 2018; Zakaria et al., 2020). The synergistic systems involve the engagement of the whole biomass-to-energy supply chain: biomethanation is optimized and harnessed as renewable energy, and natural resources such as biofertilizers are produced. However, other issues need to be addressed for its further practical development, such as energy conversion efficiency, system scale-up, reactor configuration, and economic feasibility (Huang et al., 2020; Jourdin and Burdyny, 2020; Prévoteau et al., 2020; Salimijazi et al., 2020).

**Figure 1:**
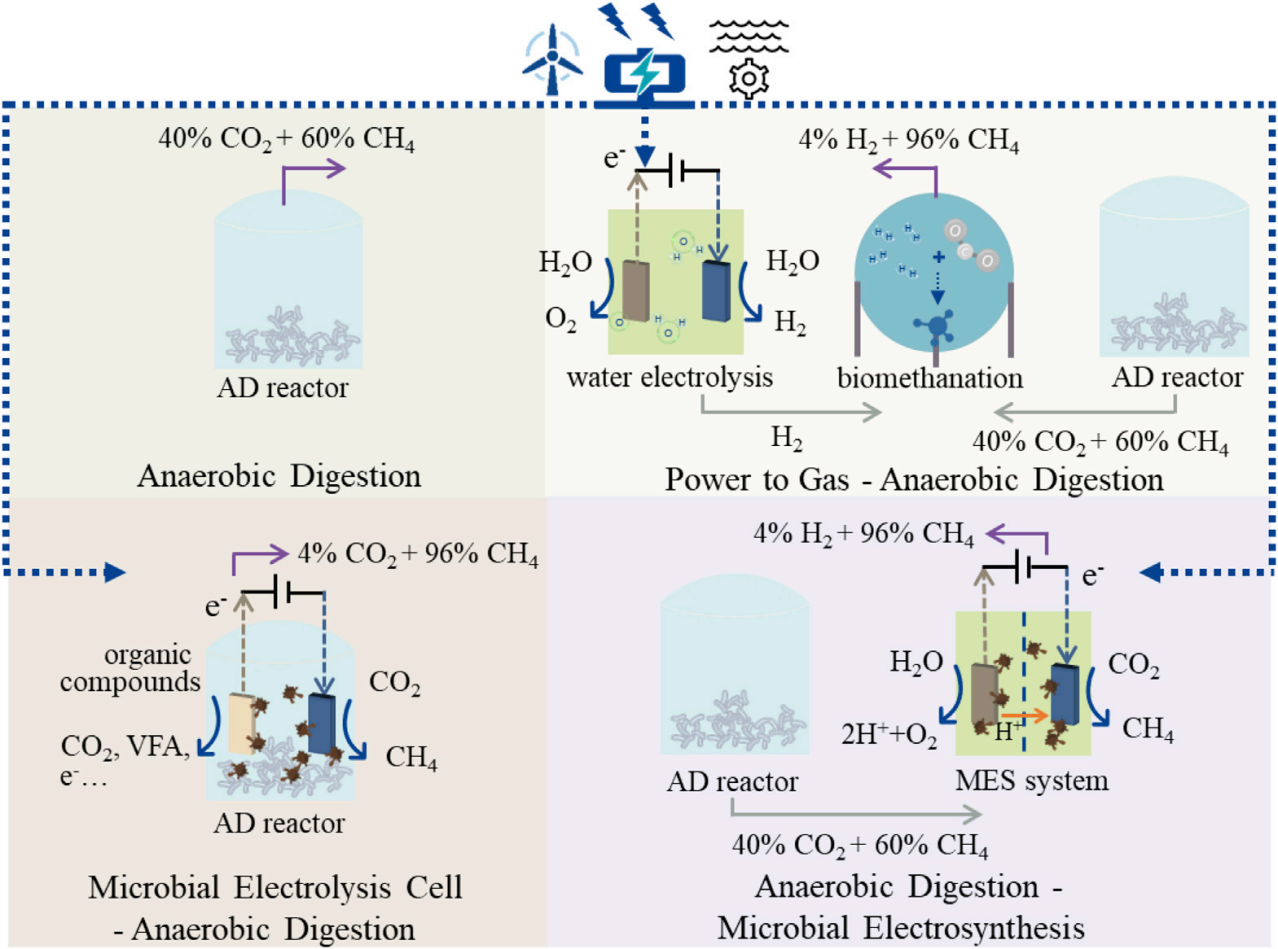
Different systems for biogas production and upgrading Three bioelectrochemical circular cascading systems with anaerobic digestion (AD) as a key platform integrated in turn with power to gas (P2G), microbial electrolysis cell (MEC) and microbial electrosynthesis (MES).

A current research gap lies in the deep understanding of potentially innovative bioelectrochemical circular cascading systems with different configurations that enable maximum biomethane production. To address this gap, this paper proposes and contrasts AD-based systems integrated with power to gas (P2G), microbial electrolysis cell (MEC), and microbial electrosynthesis (MES) (Figure 1). The advantages and challenges of the proposed systems are assessed to determine the optimal biomethane production scenario and to inform any larger-scale applications in the future. The objectives of this paper are to:

(1) evaluate sustainable feedstocks for enhanced biogas production in a temperate oceanic climate context;
(2) provide a state-of-the-art analysis of bioelectrochemical biogas upgrading technologies including P2G, MEC, and MES;
(3) provide quantitative analysis of future AD-based circular cascading systems (namely P2G-AD, MEC-AD, and AD-MES) beyond 2025 in terms of mass balance and energy return.

## Emerging biomethane production technologies

### Renewable feedstocks for AD

**Figure 2:**
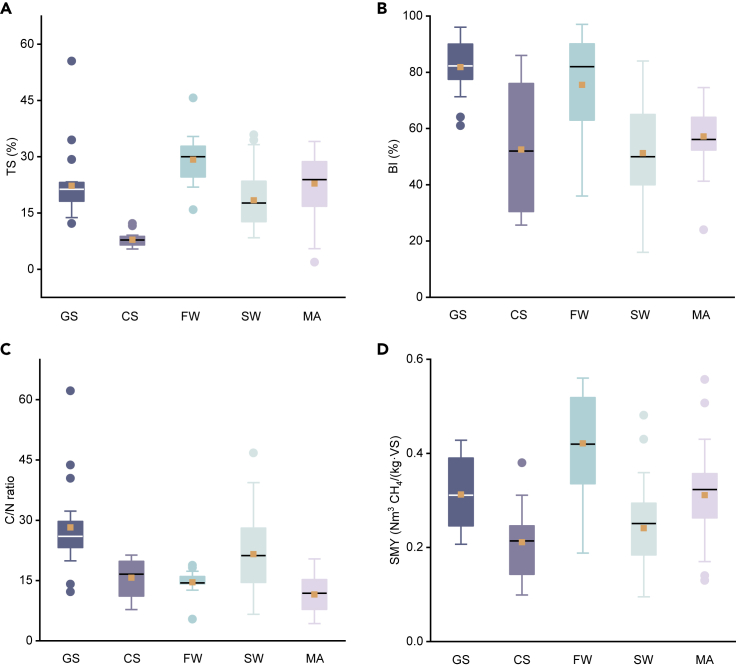
The characteristics of typical feedstocks used in anaerobic digestion The comparison of: (A) total solid (TS), (B) biodegradability index (BI), (C) C/N ratio and (D) specific methane yield (SMY) of different feedstocks, including grass silage (GS), cattle slurry (CS), food waste (FW), seaweed (SW) and microalgae (MA). The boundary of the box indicates 25^th^ percentile and 75^th^ percentile. Whiskers above and below the box range within 1.5 inter quartile range. Points above and below the whiskers indicate outliers outside 1.5 inter quartile range. The black line within the box marks the median, the solid square within the box marks the mean.

Feedstock is a crucial element in designing sustainable AD-based circular cascading systems to optimize biogas production. A variety of biomass resources can be used as the substrate for AD (Lin and Lu, 2021). Common feedstocks in a temperate oceanic climate context are mainly divided into three categories: on-farm feedstocks (such as cattle manure or slurry and energy crops such as grass or maize silage), cultivated marine feedstocks (such as seaweed and microalgae), and municipal wastes (such as food waste). The properties of different biomass feedstocks, such as total solids (TSs), volatile solids (VSs), biodegradability index (BI), specific methane yield (SMY), and C/N ratio, are critical in determining the sustainability and efficiency of biogas production (summarized in Figure 2). A complete list of analysis of feedstocks (in terms of TS, VS, BI, SMY, and C/N ratio) from the literature is included in the Data S2.

On-farm feedstocks including cattle slurry and grass silage are substrates of significant abundance in the northwest temperate oceanic climates of Europe (such as the UK and Ireland). Grassland accounted for 58.4% of total land use in Ireland in 2018 (CSO, 2020). It was anticipated that approximately 1.7 million tonnes of dry matter of grass silage could be available for AD in 2020 (McEniry et al., 2013). The biomethane yield reported for monodigestion of grass silage varies from 0.207 to 0.428 Nm^3^ CH_4_/(kg·VS) (Figure 2D). As is typical for cellulosic biomass, the major components of grass are cellulose and hemicellulose which can be easily hydrolyzed, but the degradation of recalcitrant lignin and its by-products would be a challenge for effective methanogenesis in AD. Physical and chemical properties of grass change with region, species, growth stage at harvest, conservation or fractionation methodologies, and temperature, thus it is difficult to judge all species with a unified standard. Among different varieties, perennial ryegrass is the most widespread and typical species used in many European countries, with high BI (more than 90%) in monodigestion (Himanshu et al., 2018; Wall et al., 2014a, 2014b; Wall et al., 2013). Total slurry production from all cattle in Ireland is estimated at 25.4 Mt in 2050 based on an annual production of 5.08 t/head/year and 5 million head of cattle; this corresponds to a biogas resource of 111.76 M Nm^3^, which can be further converted to 2.35 PJ of energy (Singh et al., 2010). Considering the practical operability and energetic efficiency of the AD process, monodigestion of cattle slurry is not advisable (low SMY ranging from 0.099 to 0.38 Nm^3^ CH_4_/(kg·VS), shown in Figure 2D), since the contents in cattle slurry have already passed through the livestock's digestive system and only low digestible contents are left. The median value of reported SMY of cattle slurry (0.218 Nm^3^ CH_4_/(kg·VS)) is 30.2% lower than the median value of grass silage (0.312 Nm^3^ CH_4_/(kg·VS)) in monodigestion. However, the trace elements and stabilizing buffering capacity of cattle slurry would be an advantage when codigested with other easily digestible substrates, such as grass silage (Wall et al., 2014b), food waste (Sun et al., 2020), or microalgae (Hu et al., 2021), thereby enhancing AD process stability and biomethane yield.

Food waste generation was estimated between 158 and 298 kg/person/year in the EU in 2018 by European Commission Joint Research Center (Corrado and Sala, 2018) and typically equates to one-third of the total food produced. AD is a suitable technology for treating food waste, the biomethane yield of which ranges from 0.188 to 0.56 Nm^3^ CH_4_/(kg·VS) as shown in Figure 2D. It is hard to unify the characteristics of food wastes due to the differences in geographical regions and dietary habits across the globe; however, the high content of organic matter (such as carbohydrates, fats, proteins, and other essential elements) would be a typical feature, indicating a high potential for biogas production. Compared to other types of feedstocks, food waste achieves the highest biomethane yield with a BI of ca. 97% (Figure 2B). The IEA Bioenergy Technology Collaboration Program has examined food waste digestion and concluded that it is a practical approach for energy recovery and evidence shows numerous commercial applications and potential for far greater implementation at scale (IEA, 2018). However, AD of food waste may be restricted by the inherent low C/N ratio due to the high content of nitrogen in protein. Some options for further improvement in biogas production include ultrasonic and microwave pretreatments and lipids pre-extraction (Negri et al., 2020), nitrogen removal prior to AD, and codigestion with other substrates such as cattle slurry (Sun et al., 2020) and sewage waste (Du et al., 2021), which supply additional carbon to balance the low C/N ratio.

Cultivated marine feedstocks (such as seaweed and microalgae) are categorized as advanced or third-generation biofuel sources. When compared with the second-generation biomass, the primary advantages of cultivated marine feedstocks are: the rapid growth of seaweed (macroalgae) in the marine system while sequestering CO_2_ from the atmosphere and simultaneously absorbing nutrients from (and cleansing) coastal waters; lack of requirement of arable land and lack of competition with terrestrial food and feed production. For microalgae, the optimal route to generate biomass is cultivation using captured CO_2_ from the exhaust of a biogenic process; however, low biomass yields in laboratory and full-scale cultivation present barriers to commercialization. The circularity of macroalgal and microalgal biomass through the uptake of CO_2_ through photosynthesis to mitigate carbon emissions must be noted to enhance the sustainability credentials of such feedstock. Furthermore, microalgae are rich in organic material, with the major components of carbohydrate, protein, and lipid, but no lignin content; this in theory leads to increased hydrolysis rate and biogas production efficiency as opposed to other selected feedstocks (Leong et al., 2018). Despite these advantages of algae, when used as the feedstock in AD algae have shown to present a relatively low biomethane yield (the median value of SMY of seaweed is reported as 0.251 Nm^3^ CH_4_/(kg·VS) and the median value of SMY of microalgae is reported as 0.32 Nm^3^ CH_4_/(kg·VS) as shown in Figure 2C). This is likely due to low C/N ratios (less than 15:1, ideally should be in the range of 20:1 to 35:1) (Gómez-Camacho et al., 2021) and high salinity (10.4–11.0 g/L) (Deng et al., 2020b; Tabassum et al., 2016). Ammonia inhibition induced by high nitrogen content in algae could restrict microbial growth rate and be a critical constraint in long-term continuous AD reactors, resulting in low organic loading rates, large reactor volumes and long hydraulic retention times (HRTs), and ultimately lower methane yields (Deng et al., 2020b). Co-digestion of algae with higher C/N ratio substrates, such as sewage sludge or grass silage (Ding et al., 2020; Tabassum et al., 2016), would be a cost-effective solution to generate an optimum mix for AD. To improve economic feasibility, the cost of microalgae cultivation, harvesting, and dewatering also needs to be reduced.

### Power to gas based biomethanation

The 2020 target of 20% of energy production from renewables across the EU had been almost achieved by 2019 (19.7%) according to the latest Eurostat data (Eurostat, 2020). The share of wind energy in electricity supply across the EU increased to 19.0% (559,545 GWh onshore and 129,575 GWh offshore) in 2021 compared to 14.0% (58,400 GWh onshore and 6,935 GWh offshore) in 2018 (European_Commission, 2020). Power to gas (and in particular power to methane) integrates intermittent renewable electricity with the natural gas grid and as such can maximize the use of existing energy infrastructure. Power-to-methane systems can act as a biological battery for electricity production in excess of demand. The principle of P2G-AD is to initially convert curtailed or constrained renewable electricity to hydrogen and subsequently use this hydrogen for biogas upgrading to biomethane (biomethanation) in a sequential step as per Equation 1. In a P2G system, the theoretical minimum potential difference for water electrolysis is 1.23 V. In practice, a bias voltage for anode and cathode of 0.47 and 0.3 V is applied, respectively (Liu et al., 2016a), as such this determines the practical voltage (≥ 2.0 V). The *in-situ* P2G-AD concept refers to the direct injection of the H_2_ produced from water electrolysis into an AD reactor. Biogas upgrading via *in-situ* P2G-AD may achieve a high removal of CO_2_ contained in biogas and has been shown to reach up to 99% CH_4_ purity under optimum operating conditions (Angelidaki et al., 2018). However, studies have shown several technical challenges. Firstly, hydrogen partial pressure over 10 Pa would reduce the AD system's ability to degrade volatile fatty acids (VFAs) due to the easily perturbed thermodynamic equilibrium (Liu and Whitman, 2008). As such, the microbial dynamics could be altered and the equilibrium between methanogenesis and acetogenesis could be impaired, leading to excess acidification and reduced methane production. Another issue is that continuous consumption of CO_2_ in the AD process decreases the buffer capacity, leading to an increase in pH of up to 8.5 followed by inhibition of methanogenesis. In addition, the aqueous solubility of H_2_ is rather low (0.7 mmol/L/bar in water at 55°C), which limits the gas-liquid mass transfer of H_2_, thus reducing H_2_ bioavailability and hindering the subsequent biological CO_2_ conversion.

(Equation 1) 4H_2_ + CO_2_ → CH_4_ + 2H_2_O, ΔG^0^ = − 130.7 kJ/mol (State: T = 298.15 K, pressure = 1 atm, pH = 7)

In *ex-situ* P2G-AD, biogas from AD and hydrogen from water electrolysis are used as feedstocks for enriched or pure cultures of chemoautotrophic hydrogenotrophic methanogens in a separate biomethanation reactor. In this process, CO_2_ in biogas can be converted to CH_4_ with a final CH_4_ concentration in the range of 79–98% (Table 1). Compared with the *in-situ* concept, *ex-situ* P2G-AD presents a simpler process by decoupling the biomethanation step in Equation 1 from the digestion of feedstock in the AD reactor; this segregation ensures hydrolysis and acidogenesis are not impacted by elevated hydrogen partial pressures (associated with *in-situ* biomethanation) and the stability of biogas production in AD is not affected. In the *ex-situ* concept, the external sources of CO_2_ can be diversified (such as from fermentative ethanol production) so it is more suitable for broader industrial applications (Angelidaki et al., 2018).

**Table 1:** Key parameters in *ex-situ* power to gas (P2G) upgrading process

Strategy	Temper-ature (°C)	Reactor	pH	Gas retention time, h	Gas recirculation, L/h	Maximum methane concentration, %	CO_2_ removal, %	H_2_ to CH_4_ efficiency, %	Dominant microorganisms	Reference
*ex-situ* P2G	52	two upflow reactors in series	8.5	16	4∼12	>98	∼50	<100	*Methanothermobacter thermautotrophicus*	(Kougias et al., 2017)
	52	CSTR a	8	8	4∼12	79%	∼40	60		
	52	bubble column reactor	8.3	8	4∼12	97–98	∼83	∼100		
	35	CSTR	8.17	-	-	89	69	99	hydrogenotrophic methanogens and syntrophic bacteria	(Bassani et al., 2015)
	55	CSTR	8.49	-	-	85	77	92		
	55	up-flow reactor	8.64	4-15	2.88-20.14	96	∼100	∼100	*Methanothermobacter thermautotrophicus*	(Bassani et al., 2017)
	37	biofilm plug flow reactor	-	0.24	-	98	-	-	-	(Savvas et al., 2017)
	55/65	batch	7.7-8.2	24	-	92	-	-	*Methanothermobacter*	(Guneratnam et al., 2017)

a continuous stirred-tank reactor.

To enhance the biomethanation efficiency, many approaches have been proposed, including optimizing operational conditions (such as reactor design, gas recirculation flow rate, and operating temperature), the addition of functionalized nanomaterials (Fu et al., 2020) and improved H_2_ diffusion devices that generate uniform nanobubbles (∅ 500 nm) (Rusmanis et al., 2019). The key design and operational parameters of *ex-situ* P2G-AD technology are summarized in Table 1. Low H_2_ gas-liquid mass transfer rate is still a technical challenge in *ex-situ* P2G upgrading systems which can be improved by adjusting reactor design or gas recirculation flow rate. Kougias et al. evaluated the effect of different reactor configurations (including serial upflow, continuously stirred tank, and bubble column reactors) on CO_2_ removal efficiencies and generated CH_4_ content in the output gas at more than 97% content in two upflow reactors in series and in bubble column reactors (Kougias et al., 2017). A high gas recirculation rate (12 L/h) was shown to enhance the CO_2_-to-CH_4_ efficiency (Kougias et al., 2017), mainly because the high recirculation rate increases the H_2_ retention time and maximizes the availability of H_2_ to methanogens. Increasing the operating temperature can also enable higher CO_2_-to-CH_4_ efficiency; Bassani et al. recorded a higher efficiency of CO_2_ conversion at thermophilic conditions (77%) than mesophilic conditions (69%) (Bassani et al., 2015). By setting up gas flux models rather than changing the H_2_ bubble kinetics or process intensification, Savvas et al. established a biofilm plug flow reactor, which reduced the liquid volume while increasing the gas retention time, and ultimately achieved a 98% CH_4_ conversion efficiency from CO_2_ (Savvas et al., 2017). In 2016, the world's first 1 MW P2G plant was commissioned in Denmark, demonstrating a commercially viable solution for carbon capture and reuse.

However, Partidário et al. evaluated the P2G value chain by technical and economic analysis in the Portuguese context and concluded that P2G strategy has the potential to replace conventional gas production technology only in some specific conditions, such as using endogenous resources or renewable energy sources, and improving electrolyzer efficiency (Partidário et al., 2020). In P2G applications, the common technologies include alkaline, proton exchange membrane, and solid oxide electrolysis. Electrolyzer efficiency and electrolyzer cost are key parameters influencing the commercial viability of the electrolyzer (Quarton and Samsatli, 2018). A report commissioned by “the EU Fuel Cells and Hydrogen Joint Undertaking” concluded that the strategy for future electrolyzer application should consider increasing the load factors and balancing services such as frequency response to ensure good utilization of the capital asset (Bertuccioli et al., 2014). McDonagh et al. found that electricity is by far the largest contributor (56%) to the levelized cost of energy of a P2G system (McDonagh et al., 2018), therefore, the operating cost of energy input in the form of electricity is a significant barrier to the commercialization of P2G-AD. As such, “surplus” renewable electricity with a potential low cost would be a preferrable approach to cover high electricity cost for future decarbonized energy systems.

### Microbial electrosynthesis

MES is a conversion process that uses electrical energy to generate value-added chemical products through microbial electro-catalysis (Gupta et al., 2021). Although MES is in its infancy, there is an increasing research interest in CO_2_ valorization for the production of energy dense compounds, such as carboxylic acids (including short-chain carboxylic acids, C_1_-C_5_, such as acetic and butyric acid [Christodoulou and Velasquez-Orta, 2016; Nevin et al., 2010], and medium-chain carboxylic acids, C_6_-C_12_, such as caproic acid [Prévoteau et al., 2020]), alkanes (such as methane) (Mayer et al., 2019; Nelabhotla and Dinamarca, 2019), and alcohols (such as ethanol and butanol) (Bian et al., 2020). The theory of the MES concept has been established mostly at small-scale laboratory experiments. Subsequent to the AD process, MES can function as a bioelectrochemical post-treatment method for biogas upgrading as it enables the conversion of CO_2_ to CH_4_ or value-added chemicals (such as medium-chain carboxylic acids) (Bhatt et al., 2020; Lin et al., 2021a). The AD-MES system involves two separate reactors: an AD reactor and a MES reactor. The output products from AD-MES can be flexible depending on the operation of conditions of the MES reactor (such as pH, temperature, salinity, hydrogen partial pressure, microbial inocula, and applied cathode potential) (Jourdin and Burdyny, 2020).

For a standard MES configuration, there are usually two chambers separated by a proton exchange membrane: in the anode chamber, protons or electrons are provided by the electrolytic reactions, such as the oxygen evolution reaction, chlorine evolution reaction, or biodegradation of organic material. In the cathode chamber, CO_2_ (c. 40% in biogas from conventional AD process) is reduced to CH_4_ or other chemicals (such as acetate). In the MES-AD system for biogas upgrading, CH_4_ is generated bioelectrochemically mainly via two possible mechanisms: direct electron transfer (DET) from the cathode to electroactive microbes (Equation 2), which has a lower energy barrier (E_cat_ = −0.244 V vs. standard hydrogen electrode (SHE)); or indirect electron transfer (IDET) by intermediate diffusion of H_2_ production (Equation 3), which has a higher energy barrier (E_cat_ = −0.421 V vs. SHE) but is often the main route (Gildemyn et al., 2017). Since the standard potential of the DET reaction is much higher than that of IDET hydrogen production, researchers are seeking ways to improve the energy-efficient process of DET biogas enrichment (Lin et al., 2017, 2018a, 2018b; Zhao et al., 2020). Recently Salimijazi et al. developed a theoretical calculation method on the electricity-microbes-biofuel conversion efficiency and predicted that the efficiency of engineered *in vivo* CO_2_ fixation can increase from ca. 40%–52% through DET pathway (Salimijazi et al., 2020).

(Equation 2) Direct electron transfer (DET): CO_2_ + 8H^+^ + 8e^−^ → CH_4_ + 2H_2_O

(Equation 3) Indirect electron transfer (IDET): 8H^+^ + 8e^−^ → 4H_2_, 4H_2_ + CO_2_ → CH_4_ + 2H_2_O

The two electrodes inserted in the MES reactor are the critical components with a variety of choices in terms of material, configuration, dimension, and physical pattern. The anode plays an essential role for anodic water splitting. Metal electrodes (such as Ti and Pt metal anodes) instead of carbonaceous materials (such as graphite, carbon cloth, and carbon fiber) are mostly utilized to efficiently drive oxygen evolution reaction (OER) (Kong et al., 2020). As the working electrode for CO_2_ reduction, the cathode design is of most concern in terms of the specific surface area, size, and structure (Zhen et al., 2018) (summarized in Table 2). Properties of desirable cathodes mainly include high surface area, stable surface chemical characteristics, good mechanical strength and biocompatibility (Aryal et al., 2017), all of which are targeted at cathode–microbe interaction enhancement. Some research has investigated advanced electrode configurations (such as three-dimensional [3D]-structure materials like carbon felt and carbon fiber) as well as electrode surface modification and optimization, in order to enhance microbial adhesion and electron transfer efficiency, thus maximizing the potential of MES in biogas purification (Aryal et al., 2017). For example, Li et al. reported that when the modified graphene oxide/poly (3,4-ethylenedioxythiophene) (GO/PEDOT) film carbon cloth was used as the cathode in MES converting CO_2_ to CH_4_, a 3.9-fold increase in the maximum CH_4_ production rate was recorded compared to the unmodified carbon cloth cathode; this was attributed to the modified film enhancement in the surface area in favor of microbial adhesion and biofilm formation (Li et al., 2020). A new emerging electrode configuration is the porous hollow-fiber membrane, as it can be fabricated with electroconductive carbon nanotubes, reduced graphene oxide, or metal-based catalysts which have good biocompatibility. Additionally, hollow-fiber membrane increases the cathodic specific surface area for more cathode-electroactive microbial direct interaction and enhances the electron transfer efficiency. Porous structures are provided for CO_2_/H_2_ diffusion at the cathode-microbe interface, thus solving one of the technological bottlenecks of the gas/liquid mass transfer (Alqahtani et al., 2018; Bian et al., 2018; Katuri et al., 2018). Utilization of gas diffusion electrodes is an effective approach on enhancing gas/liquid mass transfer as well. Bajracharya et al. first applied the gas diffusion electrode as a biocathode in MES supported CO_2_ reduction to multicarbon compounds (Bajracharya et al., 2016). Fontmorin et al. reported gas diffusion electrodes could enhance the bioavailability of CO_2_ and polyaniline polymer could improve the biocompatibility and conductivity of the electrodes (Fontmorin et al., 2021). Furthermore, Rojas et al. reported the highest CO_2_ dissolution (an average of 1068 mg/L inorganic carbon at 20 mL/min CO_2_ flow rate) was reached through the gas diffusion electrode (Rojas et al., 2021).”

**Table 2:** Key parameters in microbial electrosynthesis (MES) upgrading process

Strategies	Temperature (°C)	Cathode potential (V vs. SHE)	Cathode material	Current density (A/m^2^)	Coulombic efficiency (%)	Methane production rate (mmol/(m^2^ cathode area·d))	CO_2_-to-CH_4_ conversion rate (%)	Dominant microorganisms	Reference
MES	35	−0.7	graphite felt	∼0.03	92	384.3	97.7	*Methanobacterium*	(Baek et al., 2017)
	30	−0.778^a^	Pt-catalyzed carbon cloth	–	–	200	96	single Archaeon, *Methanobacterium palustre*	(Cheng et al., 2009)
	35	−0.9	carbon paper	–	85	400	76	*Methanobacterium* spp.	(Villano et al., 2010)
	25	−0.59	granular graphite	–	60	1.3^b^	60	*Methanobacterium*	(Marshall et al., 2012)
	37	−0.7	sticks of unpolished graphite	0.4	88.4	0.18^b^	>90	*Methanobacterium petrolearium*	(Xu et al., 2014)
	30	−0.7	graphite felt	1.75	95.2	210	∼95	–	(Van Eerten-Jansen et al., 2012)
	22	−0.8	porous carbon felt	–	98	603	98	*Methanobrevibacter arboriphilus*	(Dykstra and Pavlostathis, 2017a)
	22	−0.65 to −0.80	porous carbon felt with zero-valent iron	–	–	877	97	*Methanobrevibacter arboriphilus*	(Dykstra and Pavlostathis, 2017b)
	37	−1.62^a^	Ti mesh coated with Pt/C	68.07	84.81	9024	>98	*Methanobacterium*	(Zhou et al., 2020)
	30	−0.36^a^	activated carbon granules	35	67	2901.8	–	*Methanobacterium*	(Liu et al., 2018)
	35	−0.68^a^	carbon sticks	–	–	448	20.2^c^	*Methanobacteriaceae*	(Zhen et al., 2015)

a calculated based on the standard electrode potential of the Ag/AgCl (saturated KCl) reference electrode against standard hydrogen electrode (SHE) of +0.2224 V.

b the unit of methane production rate is mmol/day.

c the factors which impact low CO_2_-CH_4_ conversion rate include the configuration of MESs, the source of microorganisms, the type of membrane, the material and design of the electrodes and the distance between electrodes.

Much of the recent work on AD-MES is in pursuit of higher methane production rates and yields with high coulombic efficiency. Zhou et al. reported a maximum methane production rate of 9024 mmol CH_4_ per m^2^ projected cathode electrode surface area per day in an intact anaerobic granular sludge biocathode system, which is the highest reported rate so far (Zhou et al., 2020). Research has highlighted that hydrogenotrophic methanogens (such as *Methanobacterium*) dominate the cathodic communities in 16S rRNA gene analysis (Table 1). However, before designing the “best” AD-MES system, the underlying electron transfer mechanisms and cathode–microbe interactions need to be better understood.

Prévoteau et al. stated that the capital cost for MES, including for membranes and stable anodes for producing oxygen, is the bottleneck of MES technology (Prévoteau et al., 2020). Jourdin et al. assessed 28 important parameters for MES utilizing CO_2_ and concluded that anode material accounted for 59% of the capital cost and electricity use contributed 69% of the operating cost; both of these costs result in current MES systems being nonviable from a financial standpoint (Jourdin et al., 2020). The primary energy input is an external potential supplied to the MES reactor. For methane production, the typical external potential is usually <1.0 V in current studies (Table 1); however, MES can operate at higher voltages to produce value-added products (such as acetate and butyrate) at the cathode. It may be more economically beneficial for CO_2_ to be used for acetate production rather than CH_4_ (Christodoulou and Velasquez-Orta, 2016). Some value-added chemicals, such as short-chain organic acids including formic acid and acetic acid, could be synthesized in the MES platform by adjusting the electrode potential (Kong et al., 2020; Prévoteau et al., 2020). Different group of microbes would uptake CO_2_ to different target chemical compounds with exclusive electron selectivity by microbial modification and domestication. C_4_-C_8_ carboxylic acids can be produced by further microbial chain elongation to gain higher economic benefit (Jiang et al., 2019; Jourdin and Burdyny, 2020). Although acetic acid can be produced in MES with high selectivity (>90%) (Nevin et al., 2010), conversion of CO_2_ to longer-chain carboxylic acids would be in low specificity and at low production rates (Dessì et al., 2021). For example, Jiang et al. reported the highest production rate of caproate is 2.41 g/(L·d) (Jiang et al., 2020).

### Microbial electrolysis cell

MEC-AD works on the premise of converting organic compounds into hydrogen or methane by applying an external electric current, mainly through microbe–electrode interactions. This cutting-edge technology is of particular interest as a potential *in-situ* biogas upgrading technology. In a hybrid MEC-AD system, two electrodes are directly inserted into the AD reactor, driven by external electricity, ideally from curtailed or constrained renewable energy. The released electrons from organic matter degradation (simplified as acetate oxidation, Equation 4, E_cat_ = −0.29 V vs. SHE) at the anode can be transferred to the cathode for CO_2_ reduction, by either DET or IDET mechanism (Equations 2 and 3) (Call and Logan, 2008).

(Equation 4) Bioanode: CH_3_COO^−^ + 2H_2_O → 2CO_2_ + 7H^+^ + 8e^−^

Key parameters in MEC upgrading process including cathode material, current density, and coulombic efficiency are summarized in Table 3. Currently, the CO_2_ to CH_4_ conversion rate in bioelectrochemical systems is mainly limited by the electron supply rate (reflected by current density) and electron utilization rate by microorganisms (reflected by coulombic efficiency). The current densities reported for MES/MEC are typically around 1–100 A/m^2^ (see Tables 2 and 3). The coulombic efficiency decreases when the electron supply and consumption are imbalanced. This would be affected by several parameters such as electrode material and concentrations of electroactive bacteria. In essence, MEC-AD serves as an integrated biogas production and upgrading process, negating the need for subsequent upgrading in a separate reactor. In theory, this should lead to significant advantages for the MEC-AD system as water splitting in electrolyzers do not occur as compared to P2G-AD and a second reactor is not required as compared to AD-MES. A more detailed comparison of P2G-AD, AD-MES, and MEC-AD is provided in Table 4 in terms of the process, advantages, problems, and areas requiring improvement.

**Table 3:** Key parameters in microbial electrolysis cell (MEC) upgrading process

Strategies	Temperature (°C)	Applied voltage (V) (cathode potential (V vs. SHE))	Cathode material	Current density (A/m^3^ reactor)	Coulombic efficiency (%)	Methane production yield (L CH_4_/kg COD)	Methane production rate (m^3^/(m^3^ reactor·d))	Methane enhancement (fold)^a^	Dominant microorganisms	Reference
MEC	35	0.3	graphite pillar cathode	4.3	2.1	170.2^b^	–	1.25	–	(Feng et al., 2015)
	35	1 (−1.14)	carbon fiber brush	19.04 ± 0.29	–	408.3	–	1.3	–	(Choi et al., 2017)
	35	0.8	carbon felt	–	–	126	–	1.76	–	(Ding et al., 2016)
	10	NA (−0.68)^c^	granular activated carbon	10	60	43.4^b^	–	5.3–6.6	–	(Liu et al., 2016b)
	20	0.6	carbon cloth coated with Pt/C catalyst	–	–	207.4^b^	0.23	–	*Geobacter, Methanocorpusculum*	(Sun et al., 2015)
	25	0.8	nickel foam	–	–	196^b^	0.146	1.45	*Methanobacterium*	(Wang et al., 2020a)
	20	0.8 (−1.0)	carbon cloth covered with a Pt catalyst layer on one side	–	–	111.19^b^	0.0564	1.56	*Geobacter, Methanocorpusculum*	(Cai et al., 2016)
	30	0.8	carbon cloth with platinum catalyst	66 ± 5	81	330	0.093	–	*Methanobacteriaceae*	(Li et al., 2019)
	35	1.0 (−1.05)^c^	non-catalyzed carbon brush	–	116^d^	351	–	2.1	–	(Flores-Rodriguez et al., 2019)
	20–25	0.8	carbon cloth covered with a Pt catalyst layer on one side	–	–	–	0.138	1.64	*Geobacter, Methanobacterium*	(Liu et al., 2016c)
	35	0.3	graphite carbon mesh coated with Ni	–	–	340	0.85	1.68	*Clostridia* (class) *Methanosarcina*	(Park et al., 2018)
	35	0.3	graphite mesh coated with Ni	–	–	340	0.93	2.55	*Methanosarcina thermophila and Methanobacterium formicicum*	(Lee et al., 2017)

a the methane enhancement is compared to AD.

b assumed 1kg VS equals to 1 kg COD.

c N/A means "Not Applicable". The cathode potential is calculated based on the standard electrode potential of the Ag/AgCl (saturated KCl solution) reference electrode against standard hydrogen electrode (SHE), +0.2224 V.

d coulombic efficiency higher than 100% possibly due to oxidation of organic matter or utilization of stored energy in the microorganisms.

**Table 4:** The pros and cons of power to gas-anaerobic digestion (P2G-AD), microbial electrolysis cell-anaerobic digestion (MEC-AD) and anaerobic digestion-microbial electrosynthesis (AD-MES) bioelectrochemical circular cascading systems

	P2G-AD	AD-MES	MEC-AD
Process	• hydrogen is produced via water electrolysis; • biogas (40% CO_2_ + 60% CH_4_) from AD and hydrogen from water electrolysis are used as feedstock for enriched or pure CO_2_-type hydrogenotrophic methanogens	• biogas (40% CO_2_ + 60% CH_4_) from AD reactor is injected into a second vessel for external microbial electrosynthesis; • CO_2_ is converted to CH_4_ or other value-added chemicals at the cathode chamber.	• two electrodes are directly inserted into the AD reactor; • substrate degradation (mainly acetate) at the anode and CO_2_ reduction at the cathode.
Advantages	• biomass independent process without degradation of organic substrate • decoupled biogas and biomethane systems allow for easier process control in an *ex-situ* system • diversified external sources of CO_2_	• using electrochemically active bacteria as electrocatalysts • operational flexibility because the upgrading is occurring in a separate unit • production of diverse value-added chemicals (such as formic acid, acetic acid and butyric acid)	• easy to fabricate single chamber MEC • low-cost carbon anodes and self-sustaining microbial biocatalysts • neutral pH for microbes
Disadvantages	• low gas-liquid mass transfer rate of H_2_ • concomitant production of hydrogen and oxygen • rely on renewable and sustainable power sources	• high cost of electrode materials, membrane or separators • low specificity toward longer-chain carboxylic acids • under development	• not techno-economic feasible yet • inhibition on VFAs breakdown induced by the hydrogen partial pressure in an integrated reactor
Improvement	• reactor configuration and operating conditions (pH, CO_2_:H_2_ ratios, hydrogen partial pressure) • reduction of manufacturing and installation costs for industrial application • advanced H_2_ diffusion devices	• enhancement on direct electron transfer • development of highly biocompatible cathodes • proliferation of the electrochemical active bacteria • microbial chain elongation for more valuable products	• effectiveness of bioelectrodes • low overpotential and overall internal resistance • modification of reactor configuration • greater understanding of the microbial communities

The integrated MEC-AD system has theoretically superior potential for bioelectrochemical performance than AD-MES or P2G-AD. Firstly, MEC-AD can enhance the digestion ability within the reactor. Microbial electrolysis not only converts CO_2_ to CH_4_ but also accelerates the production rate of VFAs and finally promotes further VFAs conversion to methane. Lignocellulosic compounds are predominantly converted into easily degradable sugars by hydrolytic bacteria during AD-MEC. The derived sugars are readily available to fermentative bacteria in the subsequent acidogenesis and acetogenesis for the production of VFAs such as acetate and butyrate, the conversion of which can be facilitated by electrochemically active bacteria in MECs with a small voltage of 0.3–1.0 V (Kadier et al., 2016; Liu et al., 2005). Secondly, when compared to water electrolysis, MEC may be more efficient due to the use of a lower practical external voltage of 0.3–1.0 V (concluded from Table 3), yet the reported “best” voltage for MEC-AD varies. The reason is that the substrate, influent COD concentration, reactor configuration and electrode type vary when assessing the literature from different research groups. In the MEC-AD system, OER or direct water electrolysis is replaced by the oxidation of organic compounds at the anode. The anode potential for acetate oxidation (CO_2_/acetate, CH_3_COOH + 2H_2_O → 2CO_2_ + 8H^+^ + 8e^−^) in MECs is −0.29 V and the cathode potential for hydrogen production (2H^+^/H_2_, 2H^+^ + 2e^−^→ H_2_) is −0.41 V (Zhao et al., 2021), as such the minimum applied voltage of MECs is −0.12 V. This value is below the threshold for water electrolysis (1.23 V is the minimum electrical energy input as mentioned in the section “Microbial electrosynthesis”). In practice, more negative voltages around −0.3 to −1.0 V are employed to increase VFAs removal and gas production rates (Logan and Rabaey, 2012). Furthermore, oxygen production can be avoided in a MEC-AD system. Otherwise, oxygen would consume a substantial amount of electrons generated in AD. Therefore, without the production of oxygen, a physical separator between the anode and the cathode in an integrated MEC-AD reactor is not necessarily required. This can reduce the internal resistance and the additional energy cost for ion (such as H^+^) transfer (González-Pabón et al., 2021), thus the electron transfer efficiency and current density are improved. Lastly, the MEC-AD system may require a potentially lower capital cost due to the simple single reactor design.

Microorganisms can be inhibited or deteriorated when exposed to high electrical potential (>1.0 V) (Ding et al., 2016). The communities and growth dynamics can be altered by adding an external voltage, such as an increase in the abundance of exoelectrogens, which are more tolerant to the electrical environment that enables a higher energy conversion efficiency (Lee et al., 2017; Rousseau et al., 2020; Yu et al., 2018). The MEC-AD system is mainly dominated by hydrogenotrophic methanogenic archaea responsible for biogas production as reported in Table 3. The mechanism for the alteration in the microbial community and the improved MEC-AD performance is unclear, especially regarding the electron flows between microbe and microbe, between microbe and electrode, or between electrode and electrode. Understanding the basic principle would help design suitable electrodes and the construction of robust and efficient MEC-AD systems. Currently, increasing the biocompatible electrode surface area is a theoretical approach for enhancing biofilm formation and generating a self-assembling and self-sustaining bioelectrocatalyst. However, this approach would decrease the current density as high current density is dependent on small surface area of electrodes and good ionic conductivity. Wang et al. evaluated the effect of the surface area of a nickel foam cathode on methanogenesis in the MEC-AD system and concluded that the cathode with four nickel foam piece sheets was the most suitable to achieve the highest methane yield (Wang et al., 2020a). The reason is that the surface area of a 3D nickel foam biocathode can sustain an effective current density and improve the electroactivity of microorganisms. Other 3D-structure materials have been applied in MEC-AD reactors, for example, carbon fiber brushes of different sizes were selected to be used in a MEC-AD reactor by Baek et al., who concluded that the large surface area of carbon fiber brushes is efficient in improving MEC performance (Baek et al., 2021). The development and application of 3D-structure materials might be a breakthrough for higher methane production yield/rate in future studies.

The coupled MEC-AD reactor is a delicate and sophisticated system. A minor disturbance in process parameters such as pH, applied voltage, temperature, HRT, or substrate composition would result in bioprocess instability (Huang et al., 2020). The efficiency of the MEC-AD system can be limited by several factors: the first one is local pH variation, decreasing at the bioanode because of proton accumulation and increasing at the cathode due to proton consumption. Issues like anode acidification or cathode fouling might damage the system and destroy the biofilm (Rousseau et al., 2020). The second factor is the low hydrogen production due to the microbe–electrode electron transfer restriction and low hydrogen gas-to-liquid transfer. However, the hydrogen production could be improved by appropriate electrode design, system configuration, and operating conditions. Besides, high-efficient microorganism selection is essential in designing the MEC-AD system, since microbial interactions are complex in an integrated system. Enrichment of desirable microorganisms would be challenging and require a long adaptation period (Xafenias and Mapelli, 2014). The existing technical challenges are barriers to the development of coupled MEC-AD, as such, this technology is far from technoeconomic feasibility and still in the size of small laboratory cells.

## Comparison of AD-based circular cascading systems in four plausible future scenarios

Conventional AD systems have proven to be commercially feasible in producing biogas, yet there is room for optimization in methane purity, methane production rate, and methane production yield. Future AD systems that use circular or cascading approaches may be of benefit in optimizing technical and economic feasibility. Wu et al. evaluated the synergistic effect of biological, thermochemical (pyrolysis for production of pyrochar, pyro-oil and syngas), and P2G systems for advanced biomethane production (Wu et al., 2021). However, current research focuses primarily on improving the methane production yield/rate and technoeconomic feasibility of individual biogas upgrading technologies separately, such as P2G, MES, and MEC, while seldom comparing integrated circular cascading systems. Synergies between bioelectrochemical and AD technologies could prove beneficial in biogas upgrading, with added synergies in accelerating the degradation of VFAs, enhancing the biomethanation process and reducing CO_2_ footprint and increasing the bioenergy output (Fu et al., 2020; Huang et al., 2020; Jiang et al., 2019) through carbon capture and use. In the present study, the roles of three emerging circular cascading systems (P2G, MEC, and MES) are examined when combined with the platform technology of AD. Mass balances and energy flows are assessed to evaluate the feasibility and carbon conversion efficiency of these three bioelectrochemical systems when upgrading biogas from a variety of feedstocks. Other ancillary units such as feedstock pretreatment, water removal, and digestate treatment are outside the study's boundary. The full systems encompassing all process steps can be further studied by detailed life cycle analysis and technoeconomic analysis. The entire mass and energy flows and cycles are set up based on the assumptions specified as below:

1. The mass loading of each circular cascading system is based on 100 t fresh weight (comprised raw feedstock and water and/or returned liquid digestate) per day with different feedstocks: grass silage, cattle slurry, microalgae, seaweed, and food waste. In a normalized assessment, the TS content fed within the reactors is maintained at 8% by liquid supplementation as the suggested optimum TS contents for sustaining microbial activities in the conventional wet AD process is less than 10% TS (Shahriari et al., 2012). Extra liquor can be supplied in several ways: effluent from wastewater treatment plants, codigestion with low TS substrates, or liquid digestate from recirculation. The TS content of feedstocks affects AD performance such as metabolic products production and biogas production efficiency by resulting in a change in microbial morphology as well as microbial community structure (Wang et al., 2020b; Zhou et al., 2019).
2. Conventional AD with an amine scrubber (a mature chemical process for biogas upgrading) is considered a baseline system to compare the performance with the proposed bio-based circular cascading systems. Amine scrubbing was chosen as the baseline because of its low methane loss (<0.1%), high methane content in upgraded gas (up to 99%), and reasonable investment and operating costs (Rafiee et al., 2021). A conventional proven water electrolysis technology, alkaline water electrolysis, is adopted for the P2G system, with NaOH/KOH (liquid) electrolyte in the electrolyzer. Hydrogen generated is modeled as injected into the biomethanation system at a stoichiometric ratio of 4:1 (H_2_: CO_2_) based on the Sabatier reaction (4H_2_ + CO_2_ = CH_4_ + 2H_2_O).
3. Thermal energy consumption of each assessed system is mainly associated with heating water contained in feedstock from 10°C to 37°C (Q = cp_w_ × m × *Δ*T), where *m* (kg) represents the mass of feedstock; *Δ*T (°C) represents the difference between digester temperature (37°C) and ambient temperature (10°C); cp_w_ represents the specific heat of liquid water 4.18 kJ/(kg·°C). The specific heat of the solid portion (TS ≤ 10%) in the substrates is conservatively considered the same as that of liquid water. Electrical energy consumption mainly relates to mechanical reactions (including pumping and mixing substrates). Biogas losses and heat losses are neglected.
4. The content of final CH_4_ is supposed to be over 96% in order to meet the quality standards for green gas (EBA, 2013; IEA, 2020). Therefore, the output gas composition is assumed as 96% CH_4_ and 4% CO_2_ in the conventional AD-amine scrubber system (Bauer et al., 2013). In the P2G-AD and AD-MES system, CO_2_ conversion efficiency highly depends on H_2_/CO_2_ loading rate, H_2_/CO_2_ ratio, and retention time inside the reactor (Baek et al., 2017; Bassani et al., 2015). Moreover, unconverted CO_2_ in biogas can be potentially recirculated into the MES reactor and reutilized by microbes to achieve the targeted CO_2_ conversion efficiency. Based on the optimal operation condition, we assumed that CO_2_ in raw biogas is totally converted to CH_4_ by biomethanation in our designed P2G and MES upgrading systems, so that the upgraded biogas composition would be CH_4_ and H_2_ ignoring minor CO_2_. Therefore, the output biogas consists of 96% CH_4_ and 4% H_2_. In the MEC-AD system, since the reduction of CO_2_ at the cathode and the oxidation of organic compounds (mainly VFAs) at the anode happen simultaneously, the BI of cattle slurry and microalgae is assumed to be enhanced by 25% (Feng et al., 2015; Lin et al., 2021b). Grass silage, food waste, and seaweed are already highly biodegradable (with a BI of 90%, 86%, and 78%), so an upper limit on revised BIof 95% is imposed from a practical perspective. The output gas composition is 96% CH_4_ and 4% CO_2_ in the MEC-AD system (Li et al., 2019). The operational voltage of MEC-AD and AD-MES is assumed as 1.0 V and 3.0 V, respectively, based on literature studies (Moreno et al., 2016; Prévoteau et al., 2020).
5. The energy input for each system is divided into thermal energy consumption and electrical energy consumption. Thermal energy consumption is assumed to be provided by renewable fuels (such as wood chips) in a 90% efficient boiler. The final energy that end users consume is converted into primary energy consumption (energy consumed as input to the system) using a specific primary energy factor (PEF), to allow for comparison of input energy requirements on a primary energy basis.
6. The PEF refers to the energy conversion efficiency (inclusive for transformation and distribution losses) from primary sources (such as coal, crude oil, and other renewable energy) to a secondary energy carrier (such as electricity, fuel oil, and wood chips), which finally provides energy services to end users (EU, 2016). The PEF of wood chips used in this study is 1.1 (SEAI, 2019). PEFs of grid electricity in 2025, 2030, and 2050 are assumed to be 1.8, 1.5, and 1.0, respectively, as per EU calculation guidelines (EU, 2016).
7. The SMYs (m^3^ CH_4_/[kg·VS]) of grass silage, cattle slurry, food waste, seaweed, and microalgae are representatively selected as 0.4 (Wall et al., 2013), 0.239 (Wall et al., 2013), 0.534 (Allen et al., 2016), 0.288 (Tabassum et al., 2016), and 0.357 (Herrmann et al., 2016), respectively.

**Figure 3:**
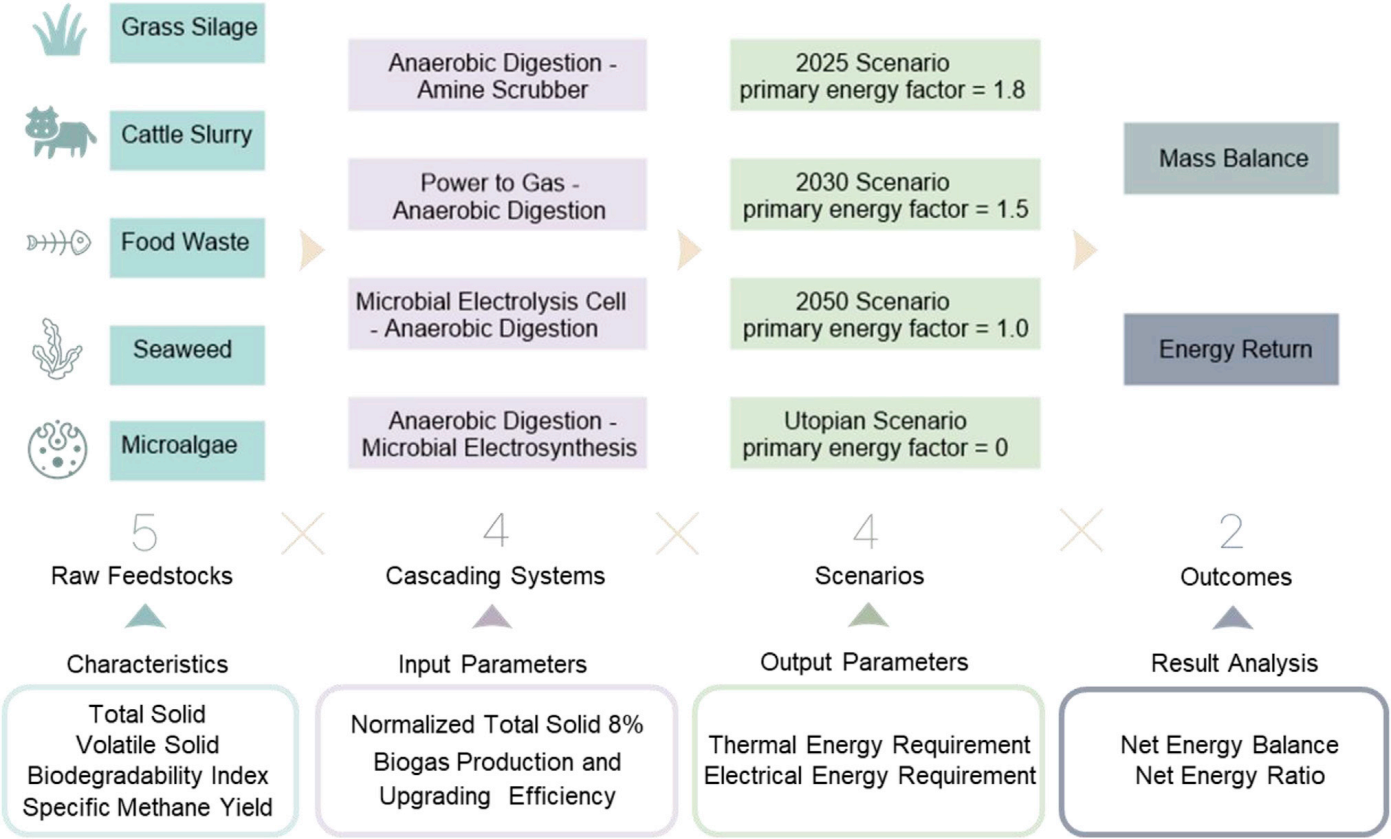
The flowchart of the methodology for the calculation of four cascading circular bio-systems

The flowchart of the methodology for calculating four cascading circular biosystems is shown in Figure 3, and the detailed calculation processes are given in the Data S1. Five different feedstocks (grass silage, cattle slurry, seaweed, microalgae, and food waste) are selected to assess the impact of substrates on four biomethane production systems: AD with amine scrubber, P2G-AD, MEC-AD, and AD-MES. Four different values of PEF are assessed to reflect the timeline of accelerated development of renewable electricity supply:

Scenario 1: PEF = 1.8 in 2025. Renewables are expected to contribute 95% of the newly installed electricity generating capacity from 2020 to 2025. IEA forecasts that renewables will account for 33% of the global electricity generation by the year 2025. With that assumption, all electrical energy consumption in our systems in Scenario 1 is assumed to be sourced from electricity with PEF = 1.8.

Scenario 2: PEF = 1.5 in 2030. With the continuing increase in electricity generation from low carbon sources (such as solar PV systems, wind, geothermal, tidal, and waves) and the driving force from the European Commission to reduce EU primary energy consumption, it is predicted that the PEF would reduce (EU, 2016). In Ireland, the Climate Action Plan 2019 states that by 2030, electricity generation shall comprise 30% nonrenewable energy resources and 70% renewable energy resources (EU, 2016). All electrical energy consumed in our systems in Scenario 2 comes from diverse electricity sources from the grid with a PEF of 1.5.

Scenario 3: PEF = 1.0 in 2050 due to the projected growth of renewable energy. The EU-27 is assumed to reach a fully sustainable energy landscape by 2050, where all grid electricity comes from available renewable energy sources, mainly solar, hydro, and wind power generation. Therefore, the final energy consumption equals primary energy consumption. In the designed systems herein, renewable energy could be stored in lower-demand periods to overcome the problem of intermittent operation, and thus, enough electricity would be provided during higher demand periods.

Scenario 4: PEF = 0 in a utopian condition. That means only constrained or curtailed electricity is provided to the proposed systems, as such the total electrical energy demand is assumed to be negligible. This can be considered as a utopian circumstance, particularly applicable during lower demand periods. In future scenarios where all electricity is renewable with a dominant portion of variable renewable electricity, there will be considerable periods of oversupply of cheap electricity. The rationale for this assumption is to explore the full energy potential of the three proposed bioelectrochemical systems.

### Mass balance

**Figure 4:**
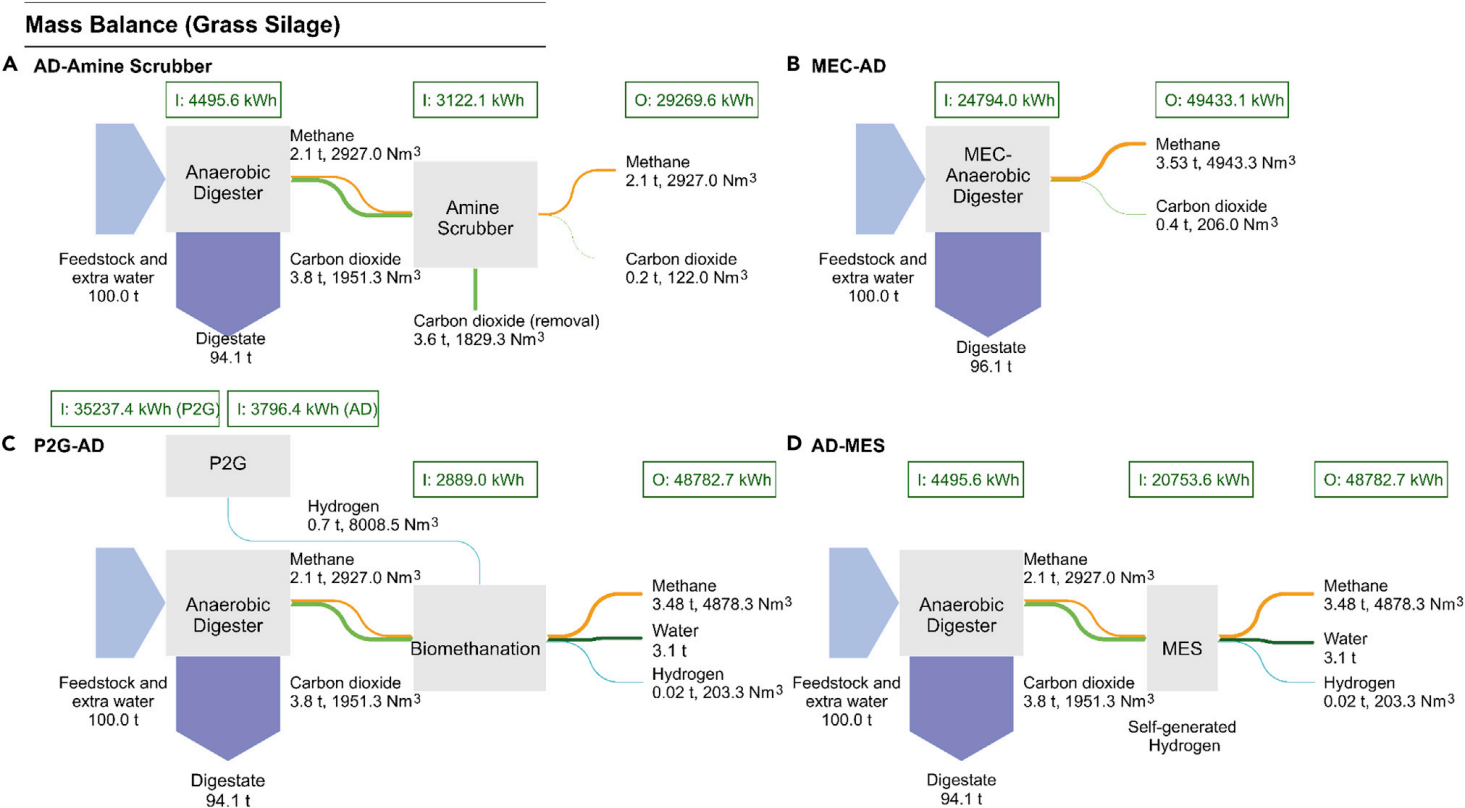
The mass balances and energy balances (based on onsite energy, PEF = 1.0) of four AD-based systems feeding 100 t fresh weight grass silage (normalized to 8% TS) per day (A–D) (A) AD-amine scrubber, (B) MEC-AD, (C) P2G-AD and (D) AD-MES. The input energy in each unit is the summation of thermal energy and electrical energy. Grass silage is adopted as the substrate. AD: anaerobic digestion; P2G: power to gas; MEC: microbial electrolysis cell; MES: microbial electrosynthesis; I energy input; O: energy output. See also Figures S1–S4.

The mass balance flow shown in Figure 4 is calculated based on the use of grass silage. The mass balances for the other four feedstocks (cattle slurry, food waste, seaweed, and microalgae) are detailed in Figures S1–S4. The performance of each system is related to the SMY of a given feedstock. For example, in the conventional AD system, 2927.0 Nm^3^ CH_4_ per 100 t fresh weight (normalized to 8% TS in digester) is produced when feeding grass silage, double the amount of methane produced by cattle slurry of 1461.9 Nm^3^ (shown in Figure S1). The reason is that the SMY (0.4 Nm^3^ CH_4_/[kg·VS]) of the selected first-cut perennial ryegrass (*Lolium perenne*) is approximately twice higher than that (0.239 Nm^3^ CH_4_/[kg·VS]) of cattle slurry. In order to compare the mass balance in different circular cascading systems, grass silage, a typical northwestern European biomass, is chosen as the representative feedstock in the following calculation.

The methane yield of the three bioelectrochemical circular cascading systems is higher than the methane yield obtained from the AD-amine scrubber upgrading system. The MEC-AD has a total methane yield of 4943.3 Nm^3^ (68.9% higher than the AD-amine scrubber upgrading system). The P2G-AD has a total methane yield of 4878.3 Nm^3^ (66.5% higher than the AD-amine scrubber upgrading system), of which 2927.0 Nm^3^ methane comes from the AD reactor and 1951.3 Nm^3^ methane is produced in the biomethanation reactor. The AD-MES has a total methane yield of 4878.3 Nm^3^ (66.5% higher than the AD-amine scrubber upgrading system) inclusive of 2927.0 Nm^3^ methane from the former AD reactor and 1951.3 Nm^3^ methane formed in the latter MES reactor (see Figure 4). The increases are due to the conversion of CO_2_ into CH_4_, which reflects the advantages of bioelectrochemical technologies in biomethane production. In the conventional AD-amine scrubber system, 94% (1829.4 Nm^3^) of the CO_2_ in biogas is captured by the amine solution, 6% CO_2_ (122.0 Nm^3^) remains in the biomethane. Therefore, the methane yield is 2927.0 Nm^3^ after going through the amine solution, equivalent to 96% of methane concentration in gas output.

The MEC-AD system has a methane yield of 4943.3 Nm^3^, which is 1.3% higher than the methane yield obtained from the P2G-AD and AD-MES systems (4878.3 Nm^3^), as a result of effective CO_2_-CH_4_ conversion within the integrated reactor. During the biogas-upgrading process, biomethanation is enhanced by both improving the feedstock's biodegradability and accelerating the microbial reaction of converting CO_2_ to CH_4_. The yield of CO_2_ in the MEC-AD system is 0.4 t, which is 89.5% lower than the CO_2_ yield obtained from AD in other three systems (3.8 t), therefore the mass of produced digestate (96.1 t) in MEC-AD is larger (around 2%) than that in other three systems (94.1 t). The nutrients concentration in dewatered sludge is going to decrease when recycling nutrients as fertilizer. Solid digestate can be used in a broad range of applications such as biofertilizer, fossil fuel replacement following combustion or pyrolysis, soil conditioner, and the raw material for biochar refining (Deng et al., 2020a, 2020b; Peng et al., 2020). Biochar can be added back to the AD reactor, in the form of electrodes or porous conductive materials, to accelerate the electron transfer process, thus enhancing methane yield in the integrated system (Escobar et al., 2021).

In the cascading system of P2G-AD biomethanation, 8008.5 Nm^3^ H_2_ is produced by alkaline water electrolysis. CO_2_ in the raw biogas reacts with H_2_ and all the CO_2_ is assumed to be converted to CH_4_ through the methanogenesis process in an *ex-situ* biomethanation reactor. Wu et al. compared the energy flow in AD-amine scrubber and P2G-AD systems and concluded that the biomethane yield enhancement by the P2G-AD is c. 70% compared to the AD-amine scrubber (60% CH_4_ and 40% CO_2_ in raw biogas) (Wu et al., 2021). Vo et al. compared the energy flow in AD-amine scrubber and P2G-AD systems and summarized that the biomethane yield of the P2G-AD system is 95% higher than the AD-amine scrubber system (50% CH_4_ and 50% CO_2_ in raw biogas) (Vo et al., 2017). The different methane yield improvement (70% vs. 95%) depends on the proportion of CO_2_ in the biogas, contributing to different methane production in biomethanation process. These results are in line with the result of an enhancement of 67% obtained in the present work. The biomethanation pathway in the AD-MES cascading system is similar to the P2G-AD system: raw biogas (60% CH_4_ and 40% CO_2_) from AD is injected into the subsequent biomethanation reactor, and CO_2_ is reacted with H_2_ generated within the reactor and converted to CH_4_. The electrolyzer is omitted and H_2_ is produced *in situ* by the hydrogen evolution reaction at the cathode. It is assumed that excess H_2_ is provided and 100% of CO_2_-to-CH_4_ conversion efficiency is achieved in P2G-AD and AD-MES systems, therefore the biomethane yields in P2G-AD and AD-MES upgrading systems are the same (4878.3 Nm^3^).

Based on the ultimate analysis of the selected grass silage (C_30_H_50_O_23_) (Wall et al., 2013), the theoretical carbon flow can be compared further for different cascading systems (calculations in the Data S1): the molar percentage of CH_4_-derived carbon as compared to the total input carbon is 46.3%, 77.2%, and 77.2% in AD-amine scrubber, P2G-AD, and AD-MES system, respectively. In comparison, MEC-AD system converts 78.2% of total carbon in 100 t fresh weight (normalized to 8% TS) to CH_4_, which shows the highest feedstock-to-CH_4_ carbon conversion efficiency. No carbon is emitted in the form of CO_2_ in the modeled P2G-AD and AD-MES system, while CO_2_-derived carbon accounts for 3.3% of effluent gas in the MEC-AD system. The above calculation provides a possible theoretical limitation of biogas upgrading efficiency of different cascading circular bioenergy systems and indicates that MEC-AD system is the most effective in terms of carbon conversion efficiency. However, the P2G-AD and AD-MES systems might achieve zero CO_2_ emission if stretched to the limit of their theoretical potential, in order to accelerate the progress in cutting EU CO_2_ emissions.

### Energy return in the form of methane based on different PEFs

**Figure 5:**
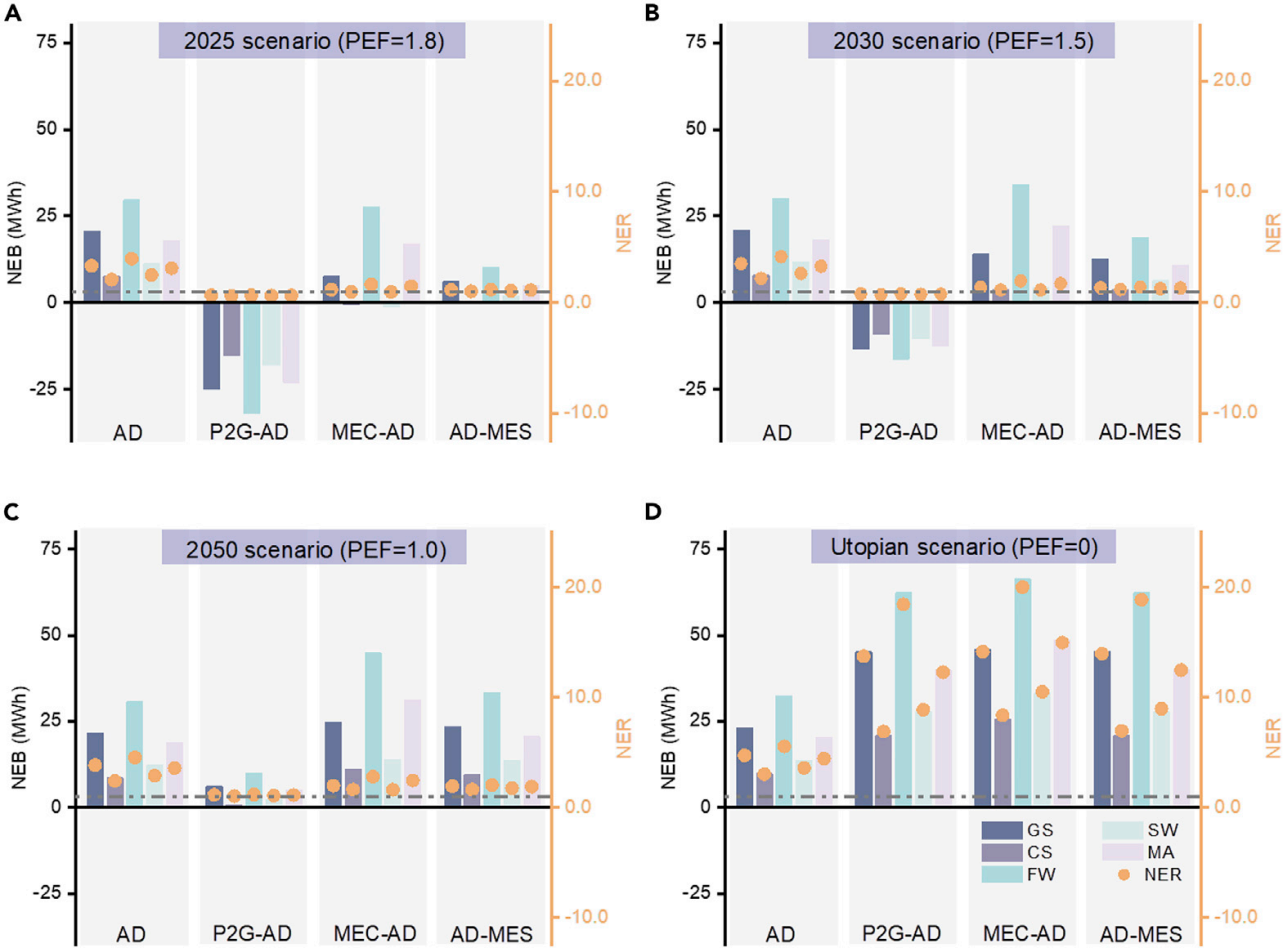
Energy return of different bioelectrochemical circular cascading systems Net energy balance (NEB) and net energy ratio (NER) of the bioelectrochemical circular cascading systems (AD, MEC-AD, P2G-AD and AD-MES) when feeding 100 t fresh weight (normalized to 8% TS) per day, in four scenarios over the next 30 years: (A) PEF = 1.8 in 2025; (B) PEF = 1.5 in 2030; (C) PEF = 1.0 in 2050 and (D) PEF = 0 in a utopian condition. The columns with different colors represent the NEB of different feedstocks including from left to right: grass silage; cattle slurry; food waste; seaweed; and microalgae. The orange solid points represent the NER of grass silage, cattle slurry, food waste, seaweed and microalgae, respectively. The dashed line represents the NER of 1. AD: anaerobic digestion; P2G: power to gas; MEC: microbial electrolysis cell; MES: microbial electrosynthesis; PEF: primary energy factor; GS: grass silage; CS: cattle slurry; FW: food waste; SW: seaweed; MA: microalgae

The circular P2G-AD, MEC-AD, and AD-MES systems are assessed in terms of energy output under different scenarios depending on the value assigned to PEF. Net energy balance (NEB) is defined as the difference between primary energy output and primary energy input. Methane is considered the only end product which produces energy. The other energy outputs inclusive of hydrogen and nutrients in the digestate are not included in the designed models but discussed more in the following section. A negative value for NEB indicates less energy is produced than the system consumes. Net energy ratio (NER) is defined as the ratio of energy output (methane in the proposed systems) divided by the energy input. An NER value of greater than 1 demonstrates a positive energy balance. The average values of NEB and NER of the five feedstocks (including grass silage, cattle slurry, food waste, seaweed, and microalgae) in each cascading biosystem are calculated in order to make a comparison between different systems. Figure 5 shows the NEB and NER of the different circular cascading bio-systems in four proposed scenarios.

In the 2025 scenario (**scenario 1**, Figure 5A), the P2G-AD system has a negative NEB (NEB –22.7 MWh, primary energy input 65.3 MWh, primary energy output 42.6 MWh, as averaged by five selected feedstocks) and an NER less than one for all feedstocks, indicating no surplus energy return would be gained in this system in a short term. The reason is that the electrical energy consumption of the electrolyzer (P2G) contributes the majority (more than 80%) of the total energy input when PEF = 1.8. For MEC-AD, a positive energy return is obtained when the feedstocks are grass silage (NEB 7.6 MWh, primary energy input 41.8 MWh, primary energy output 49.4 MWh), food waste (NEB 27.6 MWh, primary energy input 42.3 MWh, primary energy output 69.9 MWh), and microalgae (NEB 16.8 MWh, primary energy input 35.5 MWh, primary energy output 52.3 MWh). The average energy return of the AD-MES system for five different feedstocks is 4.7 MWh based on 38.1 MWh primary energy input and 42.8 MWh primary energy output, while is still 53% lower than the MEC-AD system. Amongst the bioelectrochemical circular cascading systems, MEC-AD treating food waste has the highest NER (1.7). The energy return from conventional AD combined with amine scrubber (NEB 17.3 MWh, primary energy input 8.3 MWh, primary energy output 25.6 MWh, as averaged by five selected feedstocks) is positive owing to the minor electrical energy consumption of the system.

In the year 2030 (**scenario 2**, Figure 5B), the energy return of P2G-AD (NEB –12.2 MWh, primary energy input 55.0 MWh, primary energy output 42.8 MWh, as averaged by five selected feedstocks) remains negative indicating it is still unfeasible from an energetic standpoint. However, there is an improvement in the average NEB increasing from −22.7 MWh to −12.2 MWh (46% increase) when compared to scenario 1. Primary energy consumption decreases with the variation of PEF from 1.8 to 1.5, resulting in lower primary energy input. All feedstocks treated by the MEC-AD and AD-MES system achieve positive energy returns (the MEC-AD system: NEB 15.7 MWh, primary energy input 31.8 MWh, primary energy output 47.5 MWh, as averaged by five selected feedstocks; the AD-MES system: NEB 10.5 MWh, primary energy input 32.3 MWh, primary energy output 42.8 MWh, as averaged by five selected feedstocks) at this stage as the lower PEF reduces primary energy input. Nonetheless, by reducing PEF in 2030, the impact on NEB from conventional AD-amine scrubber system is limited only experiencing a 2–3% increase. In this system, thermal energy consumption has a higher share of the total energy input (more than 70%) than electrical energy, so the variation of PEF has little effect on NEB.

In the European Energy Roadmap 2050, electricity generation in 2050 is expected to be 5141 TWh and the share of renewable electricity is set to be 83.1% (EU, 2020). Targets of supplying 100% of renewable electricity by 2050 have been set up in some countries, such as Portugal and Sweden (Yue et al., 2020). In **scenario 3** (Figure 5C), all the cascading biogas upgrading systems attain positive energy returns benefiting from the utilization of renewable electricity. In the 2050 perspective, P2G-AD is transformed from a negative energy return to a positive energy return (NEB 5.0 MWh, primary energy input 37.8 MWh, primary energy output 42.8 MWh, as averaged by five selected feedstocks) compared to the 2030 scenario, which marks the potential energetic feasibility of P2G-AD when the electrolyzer is powered by renewable electricity. The average energy return of AD-MES increases from 10.5 MWh (primary energy input 32.3 MWh, primary energy output 42.8 MWh, as averaged by five selected feedstocks) to 20.1 MWh (primary energy input 22.7 MWh, primary energy output 42.8 MWh, as averaged by five selected feedstocks) when switching from the 2030 scenario to the 2050 scenario; the average energy return from MEC-AD (NEB 25.1 MWh, primary energy input 22.4 MWh, primary energy output 47.5 MWh, as averaged by five selected feedstocks) for five feedstocks surpasses that from conventional AD-amine scrubber system (NEB 18.4 MWh, primary energy input 7.2 MWh, primary energy output 25.6 MWh, as averaged by five selected feedstocks), but when considering the ratio of energy output and input, the average NER of MEC-AD (2.1) is lower than that of AD-amine scrubber (3.4). This indicates more biomethane is generated in MEC-AD biogas upgrading system compared with AD-amine scrubber, but at the cost of consuming more electrical energy in the process.

In the utopian **scenario 4** (Figure 5D), no primary energy would be consumed by the proposed systems to produce electricity. This reflects the assumption that curtailed or constrained renewable energy may be sourced from renewable sources (such as wind, wave, and tidal energy). In Germany curtailed electricity from wind generation was 4722 GWh in 2015, with a growth rate of ca. 200%/year (Siddique and Thakur, 2020). This could possibly provide sufficient electricity for all proposed systems. For example, the most energy intensive AD-amine scrubber system consumes 21.9 GWh/year of electricity when treating food waste (100 t fresh weight food waste normalized to 8% TS per day); this is only equivalent to c. 0.5% of that total curtailment. The improvement in energy return from the AD-amine scrubber system is restricted in this scenario, with an increase in NER from 3.5 (in 2050) to 4.3. This minor increase results from the amine scrubber mainly consuming thermal energy and the primary energy demand to produce the thermal energy is not altered by a reduction of the PEF of electricity. Additionally, when compared to P2G-AD, MEC-AD, and AD-MES, the energy contained in CO_2_ is not fully utilized in the AD-amine scrubber system or converted into CH_4_ in subsequent bioelectrochemical steps. The average NEB values of P2G-AD (NEB 39.3 MWh, primary energy input 3.4 MWh, primary energy output 42.7 MWh, as averaged by five selected feedstocks), MEC-AD (NEB 44.0 MWh, primary energy input 3.5 MWh, primary energy output 47.5 MWh, as averaged by five selected feedstocks), and AD-MES (NEB 39.3 MWh, primary energy input 3.5 MWh, primary energy output 42.8 MWh, as averaged by five selected feedstocks) systems in this utopian scenario increase by more than 70% compared to the NEB values of these three systems in the year 2050. The average NEB values of the P2G-AD, MEC-AD, and AD-MES systems exceed the average NEB value of the AD-amine scrubber (NEB 19.8 MWh, primary energy input 5.8 MWh, primary energy output 25.6 MWh, as averaged by five selected feedstocks). The average NERs of P2G-AD, MEC-AD, and AD-MES (12.0, 13.6, and 12.2, respectively) are approximately three times higher than that of the AD-amine scrubber system (4.3). This indicates that the potential energy return from bioelectrochemical circular cascading systems is sizable when the primary electrical energy input is eliminated by using curtailed or constrained electricity.

**Figure 6:**
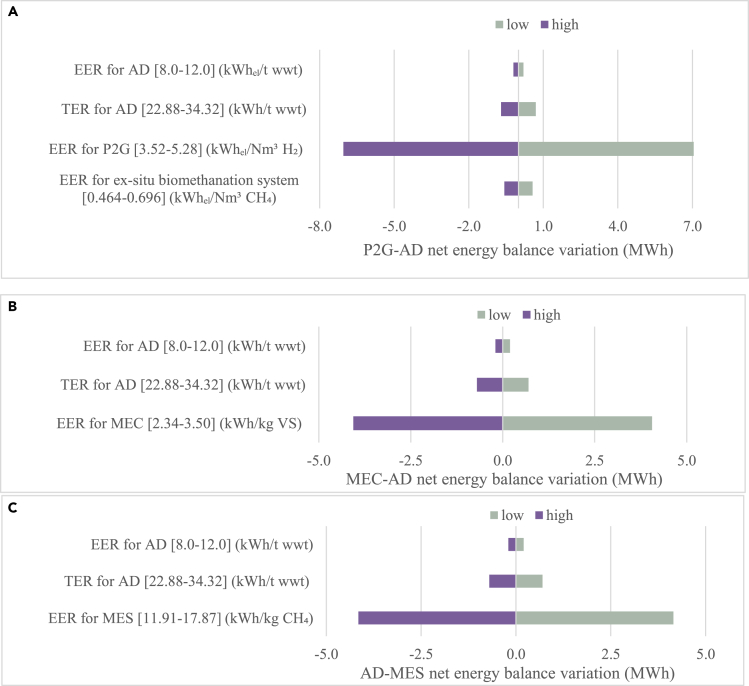
Sensitivity analysis of different bioelectrochemical circular cascading systems Sensitivity of input parameters on net energy balance, for base case analysis in (A) P2G-AD system, (B) MEC-AD system and (C) AD-MES system in scenario 3 when PEF = 1.0, feeding 100 t fresh weight grass silage (normalized to 8% TS). The variation of input parameter value is ±20%. AD: anaerobic digestion; P2G: power to gas; MEC: microbial electrolysis cell; MES: microbial electrosynthesis; wwt: wet weight; TER: thermal energy requirement; EER: electrical energy requirement.

The primary energy inputs into P2G-AD, MEC-AD, and AD-MES systems are highly influenced by the PEF value for electricity: when the PEF drops from 1.8 to 0, the primary electrical energy demand decreases leading to an increased NEB. A sensitivity analysis was carried out to assess whether the electrical energy requirement of the P2G, MEC, and MES reactors is the critical energy-consuming parameter (Figure 6). This analysis is set up in the year 2050 scenario 3 when PEF = 1.0 and when feeding grass silage (as a representative feedstock) to an AD plant. In the P2G-AD system, the NEB was most sensitive to the variation of the final electrical energy requirement for P2G. The NEB value varies by approximately 7.0 MWh (±114.4%) when the final electrical energy requirement for P2G is varied by ±20%, but varies by less than 1.0 MWh (±10%) when the final electrical energy requirement for AD or *ex situ* biomethanation system is varied by ±20%. Similar results are observed in the MEC-AD and AD-MES system: the most influential parameter influencing NEB of the MEC-AD system is the final electrical energy requirement for the MEC (NEB varying by ca. 4.1 MWh, ±16.5%); the most influential parameter influencing NEB of the AD-MES system is the final electrical energy requirement for the MES (NEB varying by ca. 4.2 MWh, ±17.6%). The variation in NEB in the MEC and MES systems follows a variation of final electrical energy requirement by ±20%. The electrical energy requirement and thermal energy requirement for AD system operation are less influential parameters. The sensitive analysis highlights that the electricity consumption of the electrodes would significantly affect the energy balance in P2G-AD, MEC-AD, and AD-MES. Therefore, in the utopian scenario 4 with no primary electrical energy consumption, the energy returns from three bioelectrochemical systems increase significantly. It indicates that to increase the energetic benefits from bioelectrochemical systems, reducing the final electricity consumption would be a rational solution.

### Limitations of the study

The numbers of biogas upgrading plants using water scrubber, membrane, and amine scrubber technology in the IEA member countries were 181, 173, and 103 in 2019 (IEA, 2019). Amine scrubbing was chosen as the baseline for conventional upgrading technology in this study; however, we acknowledge that in recent years, water scrubbing and membrane separation become mainstream biogas upgrading technologies. To reflect this mainstream change, here we compared the energy input and energy output of AD-water scrubber and AD-membrane separation with AD-amine scrubber in four scenarios (PEF = 1.8, 1.5, 1.0, 0) when feeding 100 t fresh weight grass silage normalized to 8% TS in the system (see the detailed calculation in the Data S1). The energy requirement for AD-water scrubber and AD-membrane separation is much lower than that from AD-amine scrubber in each scenario. For example, the total primary energy consumption of AD-water scrubber and AD-membrane separation in 2050 scenario (PEF = 1.0) is 5.7 MWh and 6.2 MWh, respectively, which is lower than 7.6 MWh required for AD-amine scrubber technology.

Despite the quantification on mass and energy balance of each system, there is not a straightforward answer to the question: which bioelectrochemical combination is the best? Reaction kinetics (referring to the overall biogas upgrading rates) are essential for an industrially relevant process but have not been considered in the designed models. Nonetheless, the described systems with different scenarios allow us to sketch out a technological roadmap to achieve better system sustainability beyond 2020. Taking the 2050 scenario as an example, the MEC-AD system presents the best modeled performance from an energetic standpoint (namely the highest NEB). MEC-AD has lower electricity consumption than P2G-AD and AD-MES due to the low external operational voltage (assumed as 1.0 V (Moreno et al., 2016)) for organic matter degradation. The voltage in MEC-AD is significantly lower than that required for water electrolysis (c. 2.0 V) in P2G. The presence of external voltage for the anode and cathode in MEC-AD exhibits a dual function of not only converting CO_2_ to CH_4_ but also enhancing the substrate's biodegradability, thereby leading to the improved biomethane production (Wang et al., 2021). Therefore, the achieved NER of MEC-AD is the highest modeled compared to that of the P2G-AD and AD-MES systems.

However, the MEC-AD system may face significant operational challenges in terms of achieving long-term steady biomethane production, due to the indigenous hydrogen generation in the reactor. The methane production in MEC-AD is highly reliant on the syntrophic behaviors of electroactive microbes (including fermentative bacteria and methanogens). A high voltage would accelerate the electron transfer rate and contribute to more H_2_ production, following by cathodic reduction of CO_2_ to CH_4_ by hydrogenotrophs. As the partial pressure of H_2_ increases in MEC-AD, the breakdown of VFAs could be inhibited, thereby negatively affecting the growth of hydrogenotroghs and the performance of hydrogenotrophic methanogenesis (Ding et al., 2016). Therefore, the performance of the MEC-AD system is significantly affected by internal electrochemical factors, such as the cathode material, cathode potential, and current density (Rousseau et al., 2020). The rational reactor design and precise process control are essential to ensure the optimal operation of the MEC-AD system. MEC qualifies as an “actual system completed and qualified through test and demonstration” (Zhou et al., 2018), which sits around the technology readiness level 5 (Leicester et al., 2020). It may be said that the technology is immature but the theoretical benefits provide an impetus to fund research to allow commercialization and as such optimize the integration of bioelectrochemical technologies with AD.

In the utopian scenario (scenario 4), both P2G-AD and AD-MES systems can achieve comparable performance to MEC-AD in terms of energy returns, indicating the significance of using curtailed or constrained energy. From an operational perspective, one intrinsic difference that distinguishes MEC-AD from P2G-AD and AD-MES system is that MEC-AD requires one integrated reactor, while both P2G-AD and AD-MES are sequential processes that need two reactors. Therefore, P2G-AD and AD-MES may present some operational flexibility as the complex biological process (namely AD) is separate from P2G and MES, thereby the inhibition of AD induced by a high hydrogen partial pressure can be avoided. In other words, biochemical processes of organic substrate degradation and CO_2_ biomethanation can be physically separated to ensure system stability.

Another advantage of the separate reactor configuration may be related to the intermittent nature of renewable electricity supply, particularly when using curtailed or constrained electricity. Intermittent electricity supply may alter or damage the function of electroactive microbial communities in the MEC-AD reactor, thus inhibit the syntrophic production of biomethane (Rousseau et al., 2020). In comparison, intermittent electricity supply may not severely affect the efficiency of water electrolysis in P2G. P2G and MES can be used either alone or combined with AD to produce desired products. In addition to the CO_2_ in biogas, other external sources of CO_2_ such as from food and beverage and cement industries can also be used to increase the system flexibility and capacity (Bassano et al., 2019). It must be noted that although other energy outputs inclusive of hydrogen and nutrients in the digestate are outside the study's boundary, they can be recovered as by-products to build more sustainable circular economy. Furthermore, a wide range of targeted products (such as methane, alkanes, alcohols, and carboxylic acids) could be produced through the use of versatile biocatalysts in MES, and the production of C2−C6 monocarboxylic acids by MES has been demonstrated at technology readiness level of 1–3 (Dessì et al., 2021; Jourdin and Burdyny, 2020; Lin et al., 2021a).

## Conclusions

Three future bioelectrochemical circular cascading systems with different configurations (P2G-AD, MEC-AD, and AD-MES) have been modeled and assessed in terms of the potential to reduce carbon emissions and maximize biomethane production. Mass flows show the highest modeled methane production (4943.3 Nm^3^ per 100 t fresh weight grass silage normalized to 8% TS) in the MEC-AD system compared to the P2G-AD and AD-MES systems, possibly due to the potential for an efficient CO_2_-to-CH_4_ conversion in a single integrated reactor. By the year 2050, when all electricity is proposed to come from renewables, the MEC-AD system is modeled as presenting the highest NEB value (NEB 25.1 MWh, primary energy input 22.4 MWh, primary energy output 47.5 MWh, as averaged by five selected feedstocks) compared to the conventional AD-amine scrubber (NEB 18.4 MWh, primary energy input 7.2 MWh, primary energy output 25.6 MWh, as averaged by five selected feedstocks). However, the theoretical maximum energy return of the MEC-AD system can only be achieved in a highly elaborate reactor at the limit of its potential. In contrast, the P2G-AD and AD-MES systems employ two reactors each for easier operation and control. P2G-AD, MEC-AD, and AD-MES cascading biosystems all gain positive energy returns when the electricity source would otherwise have been curtailed or constrained as defined by a PEF of zero. The electricity source is postulated to be the fundamental limitation on the sustainable commercial application of P2G-AD and AD-MES.

This analysis aids in the decision process of how best to integrate the electricity grid into the production of advanced biofuels. Nonetheless, much remains to be optimized to bring these emerging bioelectrochemical technologies to possible industrial application. For example, a high applied voltage would be required to overcome the overpotential which means more input energy consumption than the theoretical requirement. Furthermore, the instability of microbial communities under an external voltage during long-term operation might result in sub-optimal reactor performance and low production rates of end-products in MES/MEC for an industrial process. Ultimately, both out of the box thinking and solid evidence of successful bioelectrochemical cascading circular systems are required to approach competitiveness in biogas production and upgrading.

## Supplemental information

Document S1. Figures S1–S4

Data S1. Mass flow and energy balance calculation.xlsx, related to Figures 4 and 5

Data S2. Feedstock characteristics
