# Supplementary material for: Emerging bioelectrochemical technologies for biogas production and upgrading in cascading circular bioenergy systems
Source: iScience. 2021 Aug 18;24(9):102998. doi: 10.1016/j.isci.2021.102998 (PMC8426204; doi:10.1016/j.isci.2021.102998)
Supplement: Document S1. Figures S1–S4 [file mmc1.pdf]

iScience, Volume *24*

Supplemental information

Emerging bioelectrochemical technologies for biogas production and upgrading in cascading circular bioenergy systems

Xue Ning, Richen Lin, Richard O'Shea, David Wall, Chen Deng, Benteng Wu, and Jerry D. Murphy

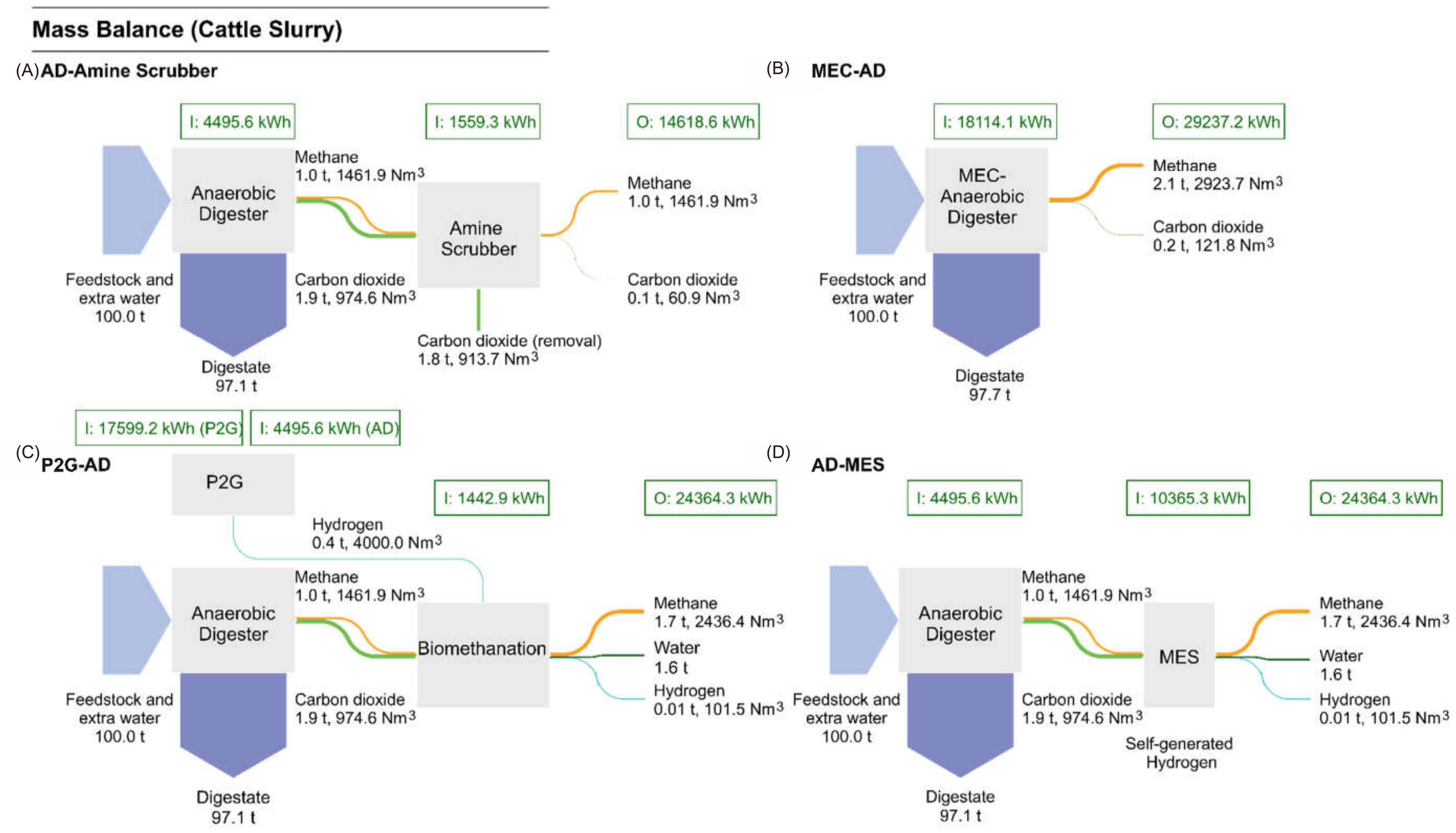

Fig. S1. The mass balances and energy balances (based on onsite energy, PEF = 1.0) of four AD-based systems feeding 100 t fresh weight cattle slurry (normalized to 8% TS) per day: (A) AD-amine scrubber, (B) MEC-AD, (C) P2G-AD and (D) AD-MES, related to Fig. 4. The input energy in each unit is the summation of thermal energy and electrical energy. AD: anaerobic digestion; P2G: power to gas; MEC: microbial electrolysis cell; MES: microbial electrosynthesis; I: energy input; O: energy output

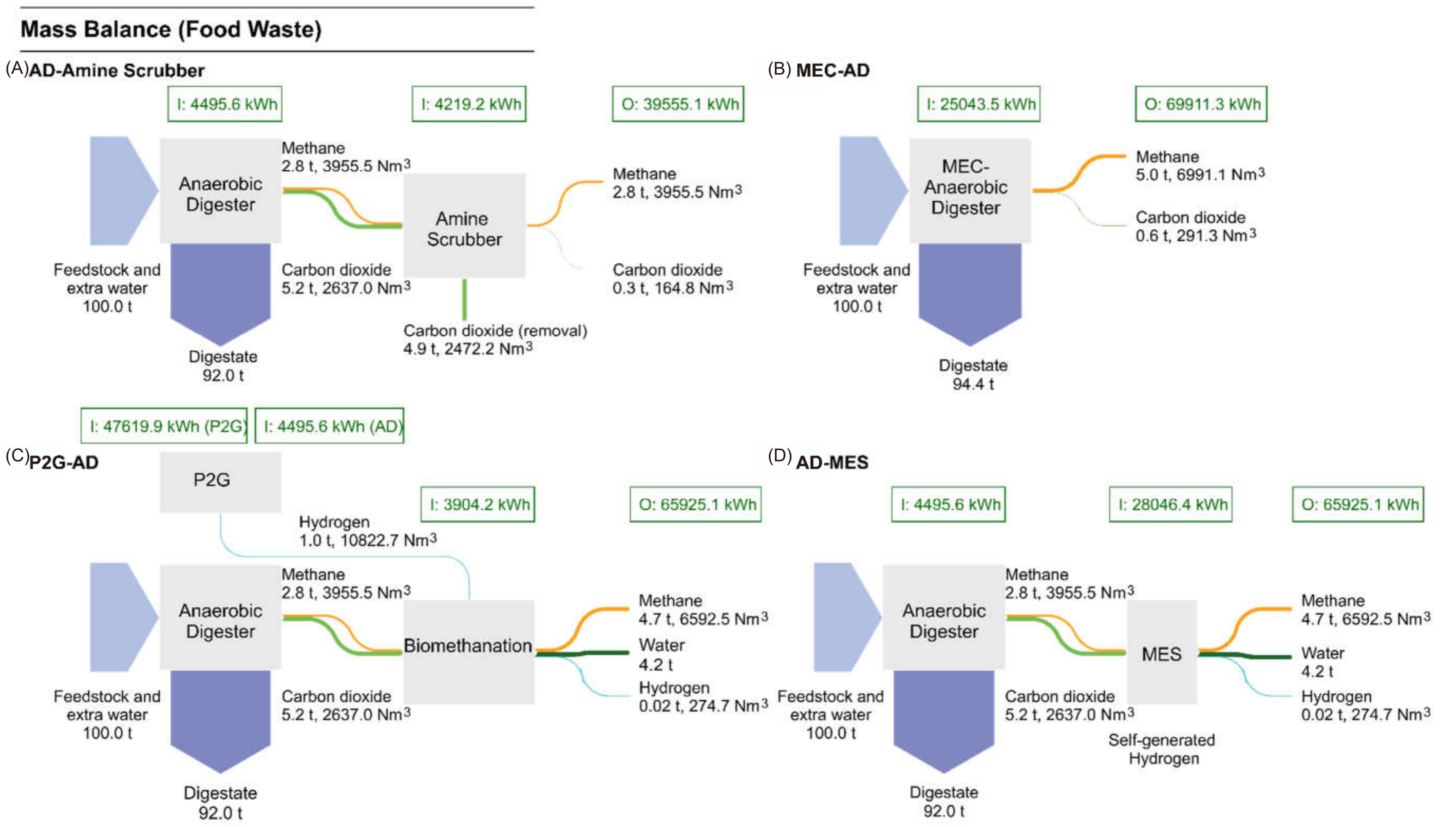

Fig. S2. The mass balances and energy balances (based on onsite energy, PEF = 1.0) of four AD-based systems feeding 100 t fresh weight food waste (normalized to 8% TS) per day: (A) AD-amine scrubber, (B) MEC-AD, (C) P2G-AD and (D) AD-MES, related to Fig. 4. The input energy in each unit is the summation of thermal energy and electrical energy. AD: anaerobic digestion; P2G: power to gas; MEC: microbial electrolysis cell; MES: microbial electrosynthesis; I: energy input; O: energy output

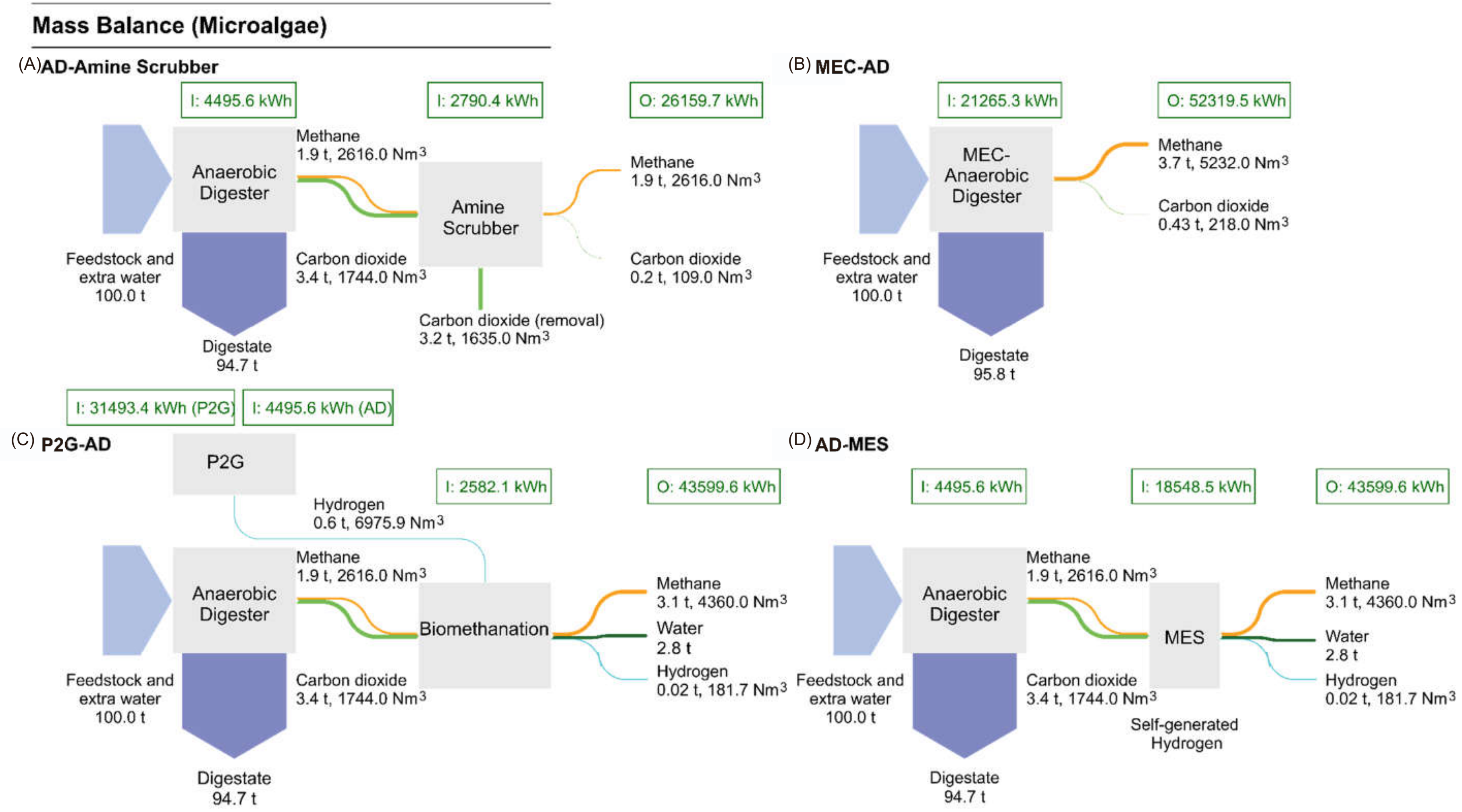

Fig. S3. The mass balances and energy balances (based on onsite energy, PEF = 1.0) of four AD-based systems feeding 100 t fresh weight microalgae (normalized to 8% TS) per day: (A) AD-amine scrubber, (B) MEC-AD, (C) P2G-AD and (D) AD-MES, related to Fig. 4. The input energy in each unit is the summation of thermal energy and electrical energy. AD: anaerobic digestion; P2G: power to gas; MEC: microbial electrolysis cell; MES: microbial electrosynthesis; I: energy input; O: energy output

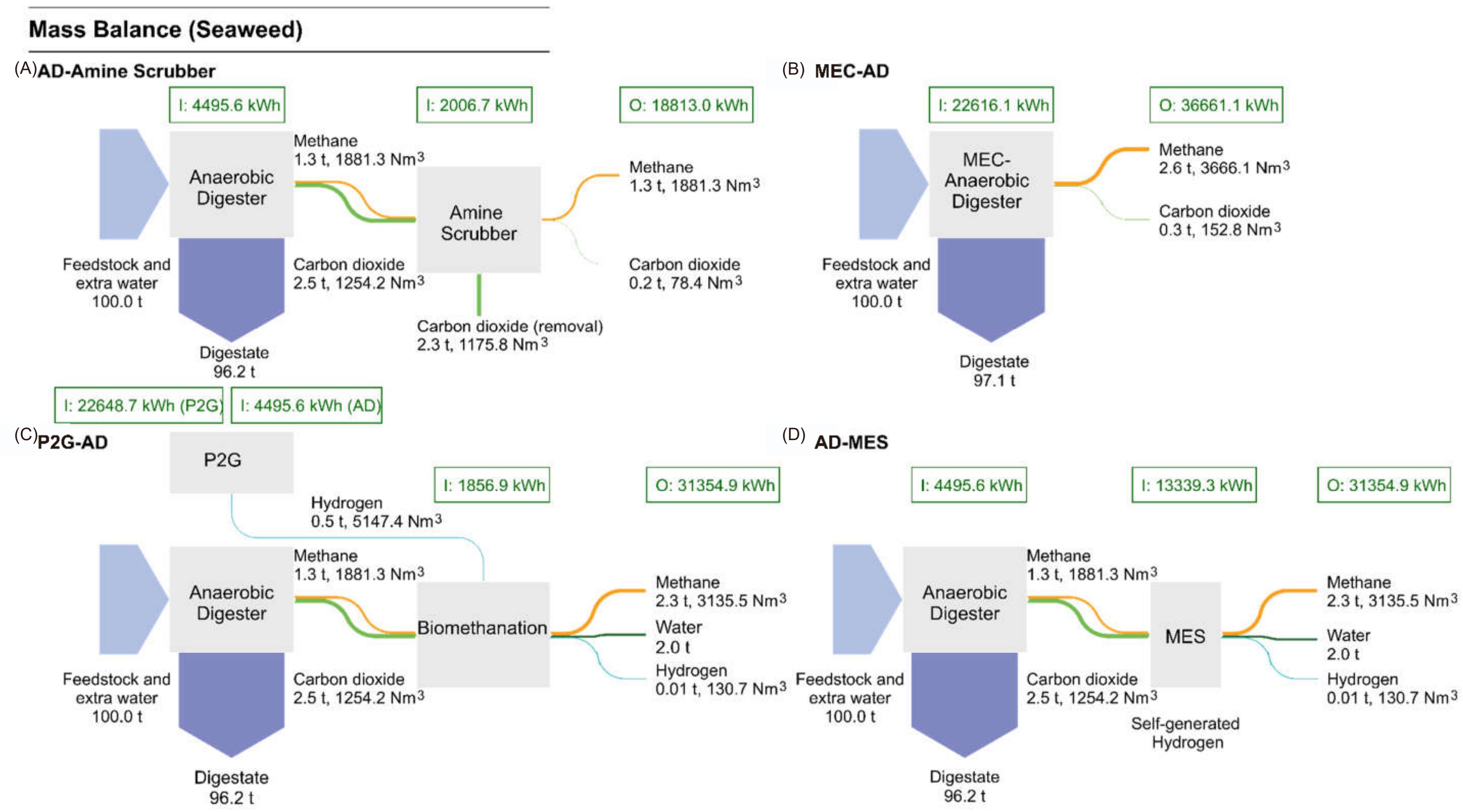

Fig. S4. The mass balances and energy balances (based on onsite energy, PEF = 1.0) of four AD-based systems feeding 100 t fresh weight seaweed (normalized to 8% TS) per day: (A) AD-amine scrubber, (B) MEC-AD, (C) P2G-AD and (D) AD-MES, related to Fig. 4. The input energy in each unit is the summation of thermal energy and electrical energy. AD: anaerobic digestion; P2G: power to gas; MEC: microbial electrolysis cell; MES: microbial electrosynthesis; I: energy input; O: energy output
